# Cavitation based cleaner technologies for biodiesel production and processing of hydrocarbon streams: A perspective on key fundamentals, missing process data and economic feasibility – A review

**DOI:** 10.1016/j.ultsonch.2022.106081

**Published:** 2022-06-24

**Authors:** Elvana Cako, Zhaohui Wang, Roberto Castro-Muñoz, Manoj P. Rayaroth, Grzegorz Boczkaj

**Affiliations:** a Department of Process Engineering and Chemical Technology, Faculty of Chemistry, Gdańsk University of Technology, Poland; b Shanghai Key Lab for Urban Ecological Processes and Eco-Restoration, School of Ecological and Environmental Sciences, East China Normal University, Shanghai 200241, China; c Institute of Eco-Chongming (IEC), No.20 Cuiniao Road, Chen Jiazhen, Shanghai 202162, China; d Technology Innovation Center for Land Spatial Eco-restoration in Metropolitan Area, Ministry of Natural Resources, 3663 N. Zhongshan Road, Shanghai 200062, China; e Department of Sanitary Engineering, Faculty of Civil and Environmental Engineering, Gdańsk University of Technology, Poland; f Tecnologico de Monterrey, Campus Toluca. Av. Eduardo Monroy, Cárdenas 2000 San Antonio Buenavista, 50110 Toluca de Lerdo, Mexico; g GREMI, UMR 7344, Université d'Orléans, CNRS, 45067 Orléans, France; h EkoTech Center, Gdansk University of Technology, G. Narutowicza St. 11/12, 80-233 Gdansk, Poland

**Keywords:** Cavitation, Fuels, Biodiesel, Desulfurization, Organic synthesis, Waste management, HC, hydrodynamic cavitation, AC, acoustic cavitation, C_v_, cavitation number, TAC, thermally activated carbon (thermic activated clay), Shirasagi TAC, trade name for a commercial activated carbon, CFP-450, carbon adsorbent, synthesized from Cassia Fistula biomass at 450°C, CFP-450-Ni, nickel impregnated carbon adsorbent, CFP-450-Cu, copper impregnated carbon adsorbent, CFP-450-Ni-Cu, double metal impregnated carbon adsorbent, TAC-Zn, zinc impregnated activated carbon, TAC-Co, cobalt impregnated activated carbon, TAC-Ni, nickel impregnated activated carbon, TAC-Ni-Cu, double metal impregnate activated carbon, 4,6-DMDBT, 4,6- dimethyl dibenzothiophene, DBT, dibenzothiophene, BT, benzothiophenes, MEK, methyl ethyl ketone, API, API gravity (American Petroleum Institute), CFD, computational fluid dynamics, CV, Circular Venturi, OP1, single hole orifice, OP2, rectangular slit orifice, OP3, 100 holes orifice plate, FAME, fatty acid methyl ester, TLIM, Lipozyme TLIM, trade name of Thermomyces lanuginosus, SAGD, steam-assisted gravity drainage, HFO, heavy fuel oil, MOF, metal organic framework, TMU-17-NH_2_, an amino-functionalized Zn-based MOF, PTA, phosphotungstic acid. PTA@MIL-53 (Fe), phosphotungstic acid encapsulated iron-based metal framework, Tween 80 and Span 80, surfactants used in section 6 processes

## Abstract

The present review emphasizes the role of hydrodynamic cavitation (HC) and acoustic cavitation in clean and green technologies for selected fuels (of hydrocarbon origins such as gasoline, naphtha, diesel, heavy oil, and crude oil) processing applications including biodiesel production. Herein, the role of cavitation reactors, their geometrical parameters, physicochemical properties of liquid media, liquid oxidants, catalyst loading, reactive oxygen species, and different types of emulsification and formation of radicals, formation as well as extraction of formed by-products are systematically reviewed. Among all types of HC reactors, vortex diode and single hole orifices revealed more than 95 % desulfurization yield and a 20 % viscosity reduction in heavy oil upgrading, while multi-hole orifice (100 holes) and slit Venturi allowed obtaining the best biodiesel production processes in terms of high (%) yield, low cost of treatment, and short processing time (5 min; 99 % biodiesel; 4.80 USD/m^3^). On the other hand, the acoustic cavitation devices are likely to be the most effective in biodiesel production based on ultrasonic bath (90 min; 95 %; 6.7 $/m^3^) and desulfurization treatment based on ultrasonic transducers (15 min; 98.3 % desulfurization; 10.8 $/m^3^). The implementation of HC-based processes reveals to be the most cost-effective method over acoustic cavitation-based devices. Finally, by reviewing the ongoing applications and development works, the limitations and challenges for further research are addressed emphasizing the cleaner production and guidelines for future scientists to assure obtaining comprehensive data useful for the research community.

## Highlights

• Cavitation phenomenon increases the effectiveness of conventional processes.
• Effective technologies for hydrocarbons (gasoline, diesel, naphtha) processing.
• Cavitation is useful in biodiesel production.
• Cost effective desulfurization of fuels by cavitation and oxidation.
• Proved usefulness for heavy oil upgrading.

## Graphical abstract

**Figure:**
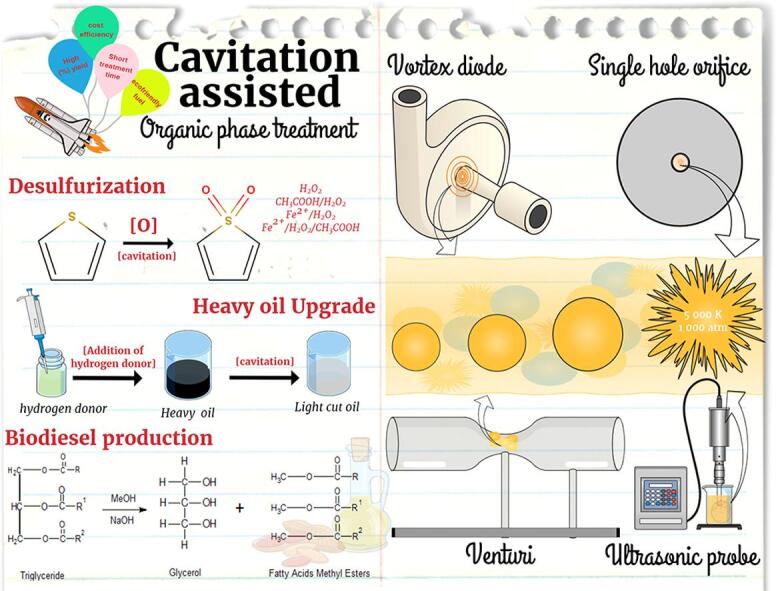

## 1 Introduction

The depletion of energy resources affects world’s economic sustainability. The rapid growth in the population and industrialization leads to the continuous and extremely use of the existing energy resources. Presently, fossil fuels, oil, coal and gas are the major sources of energy [1], in which, for example, petroleum represents the primary and leading energy resource all over the world [2]. It is known that crude oil is a natural resource of petroleum and it supplies fuel for transportation and chemical industries. It is divided into light crude oil and heavy crude oil, which are both needed to meet the growing fuel demand of society and industries. Importantly, due to its low viscosity, high API gravity, and low concentration of hetero-atoms, the oil refineries strongly depend on the light crude oil for the production of gasoline and diesel fuel. As a non-renewable energy source and the continuous use of the light oils, their availability is steadily decreased [3]. On the contrary, heavy crude oil is widely available but its processing involves a significant cost along with environmental issues [4]. The main representative of global hydrocarbon reserves is the heavy oil or bitumen, which is characterized by high asphaltene content, high viscosity, low API gravity, high heteroatom (S, N, and O) and heavy metals content [5]. The petroleum refineries have emphasised the utilisation of suitable techniques to upgrade (convert) heavy oil fractions into the lighter ones, and other high value-added products [[6], [7], [8]]. To date, several conventional techniques, such as carbon rejection, hydrogen addition processes, and separation processes, have been proposed for the upgrading of heavy oils. The conventional processes usually treat atmospheric residues, but they require a good quality of feedstocks, high catalyst consumption and unit operability. The SOx emission associated with the fuel processing represents a serious environmental issue over the last years [9]. A variety of approaches focuses on the deep desulphurization processes that would offer the minimization of Sulfur content from these fuels to an acceptable limit. Even though is effective in the removal of S content, the appropriate reactor design in the catalytic desulphurization affects the economic viability of the process and the catalyst dose, and their instability reduces the fuel quality [[10], [11]].

The aforementioned energy sources emit different kinds of toxic gases, such as SOx, carbon dioxide, along with particulate matter and other gases, to the atmosphere and many other toxic pollutants to the environmental matrices [[12], [13], [14]]. At this point, biodiesel is the next alternative fuel, which is mainly produced from vegetable oils, animal fat, and recently by recycling waste cooking oil. It is ascertained that biodiesel, in contrast to petroleum-based fuels, is a promising and environmentally friendly option [[15], [16]]; unfortunately, it demands more complex processing, especially concerning waste sources [[17], [18]]. Therefore, cleaner technologies with potential economic viability have been adapted for the rapid production of fuels in both petroleum refining processing and renewable biodiesel production. This paper highlights an emerging technique, such as cavitation, in the fuel processing applications, as well as for the transesterification process for biodiesel production.

The cavitation process has been applied in a number of applications in various fields, including wastewater treatment [[19], [20]], chemical processing [21], biotechnology [22], polymer chemistry [23], textile industry discharges, petroleum industries [[24], [25]], among others. For instance, hydrodynamic cavitation (HC) and sonocavitation belong to the group of technologies that ensure safer and more energy-efficient systems [26]. Apart from the broad application of these technologies in wastewater treatment, they were found appropriate even in organic phase processes, such as heavy oil upgrading, desulfurization, denitrogenation processes of fuels and biofuel production.

To date, most of the reviewed papers are related to the cavitation phenomenon that takes place in the aqueous phase. Processes based on cavitation, specially dedicated to wastewater treatment, have been well explored, however, there are still many aspects to be deeply studied, e.g., an in-depth analysis of the advantages and applications of the cavitation phenomenon in the organic phase is scarce. Therefore, a detailed literature search on this topic was done and reviewed. In this review, the highlighted applications of cavitation on organic phase treatment, such as crude oil processing [27], biodiesel production [28], desulphurization of fuels [29], improving rheological characteristics of oils [30], treatment of heavy oils, hydrocarbon cracking [31], and emulsion production during cavitation processes [32], are addressed. The upgrading of heavy molecules in residue, or other heavy cuts, can be done by cavitation technology but under specific operating conditions [33]. The research studies published so far indicate that cavitation may be useful for the improvement of the properties of crude oil, diesel, gasoline, naphtha, and waste cooking oil toward more friendly products to the environment and more beneficial from the economic point of view.

During organic phase treatment aided by cavitation, occasionally a second – aqueous phase is added in the form of oxidants which favours the formation of emulsions. This becomes a significant aspect impacting the yield of some processes [34], where the presence of emulsion results in a high interfacial area and increased mass transfer rate. Therefore, one of the goals of this review was to set on point the influence of HC and acoustic cavitation in various processes associated with chemical transformations taking place in the organic phase medium. The role of emulsification in (%) yield of selected processes of organic phase treatment under cavitation conditions is discussed, along with a comparison in terms of methodology and estimated cost of cavitation processes.

**Fig. 1:**
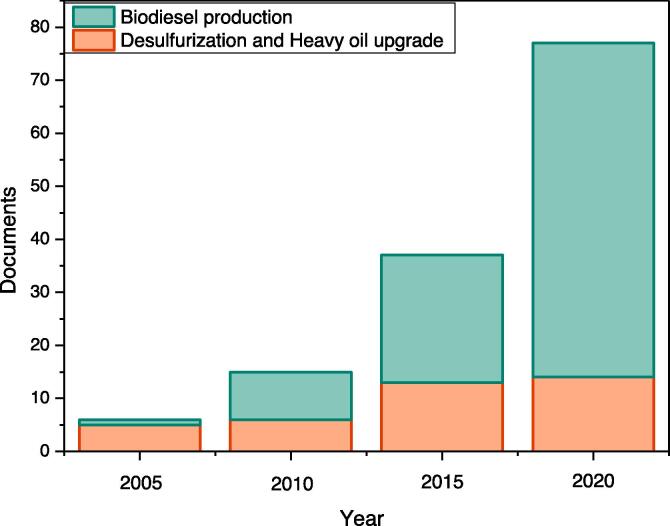
Number of studies on the processing of hydrocarbon streams aided by cavitation (source: Scopus.com/ 2021).

As can be seen in Fig. 1, there are more studies conducted on the application of cavitation in biodiesel production, compared with the number of studies investigating the desulfurization of fuel and heavy oil upgrade. All these applications are addressed well in the following sections.

## 2 Principles of cavitation phenomenon and types of cavitation

Cavitation is described as a decrease of the pressure in the liquid media responsible for the generation of cavities, whose size keeps increasing until reaching the absolute magnitude and the cavity implodes [35]. The high temperature (in the range of 5,000 – 10,000 K) and pressure (500 atm) inside the bubble ensure the right conditions to destroy strong carbon chains present in different kinds of pollutants [36]. Hence, destruction/removal of contaminants in the presence of cavitation is described as two routes possible mechanisms, which are depicted in the cavitation phenomenon as free radical attack and pyrolysis [37].

HC and acoustic cavitation (AC), are the major cavitation methods for various chemical/physical transformations in the liquid media. In acoustic/sonocavitation bubbles are generated by the high-frequency sound wave and in HC pressure drop of the pumped fluid is caused by a geometrical constriction generating bubbles. [[38], [39]]. Cavitation processes are strongly depending on several factors such as (i)Physicochemical properties of pumped media (including the viscosity of treated media, density, vapor pressure, surface tension, presence of other compounds like surfactants), (ii) Control of process parameters, such as pH, temperature, inlet pressure, the concentration of pollutants, treatment time, molar ratio of pollutant and oxidants and (iii) the geometry of the cavitating device.

Therefore, the challenges involved in organic phase treatment using cavitation are, i). high vapour pressure liquids obtain reduced cavitation intensity [40], ii) The collapse intensity of cavities in organic phase media is weaker compared to water phase due to high vapour pressure and low surface tensions of organic liquids, which could be explained by Rayleigh Plesset equation (**Eq.** (1)) [26].

(1) $$\frac{pB\left(t\right)p_{\infty }\left(t\right)}{\mathrm{pL}}=R\frac{d^{2}R}{d^{2}}+\frac{3}{2}{\left(\frac{\mathrm{dR}}{\mathrm{dt}}\right)}^{2}+\frac{4v_{L}}{R}\frac{\mathrm{dR}}{\mathrm{dt}}+\frac{2S}{p_{L}R}$$

where R is the radius of the bubble, p∞(t) is the pressure far from the bubble, ρ_L_ is the liquid density, ν_L_ is the dynamic viscosity, and S is the surface tension.

iii) The viscosity is another physicochemical parameter influencing the treatment effectiveness of the organic phase [[41], [42]].

iv) The geometry of the cavitating device is another key factor in the cavitation inception. The number and diameter of holes, thicknesses of the orifice plate, and total flow area indicate the effectiveness of the organic phase processes in HC. One geometrical parameter related to the number of holes and their hole diameter is written as ‘α’ which is expressed as a ratio of the total perimeter of holes to the total flow area of the plate, as denoted in **Eq** (2). It describes that as the number of holes increases, the ‘α’ parameter gets bigger and, as a result, higher (%) yields will be achieved [36]. ‘β’ is another geometrical parameter that is less used but it describes the cavitation intensity as a function of the device geometry. ‘β’ is defined as the ratio of the sum of the hole areas over the cross-sectional area of the pipe, as denoted in **Eq** (3). Both geometrical parameters (i.e., ‘α’ and ‘β’) give an insight into the generated cavities and the intensity of collapse between them [43].

(2) $$\alpha =\frac{\mathrm{Totalperimeterofholes}}{\mathrm{Totalflowareaoftheplate}}\left({\mathrm{mm}}^{-1}\right)$$

(3) $$\beta =\frac{\mathrm{Sum}\;\mathrm{of}\;\mathrm{the}\;\mathrm{hole}\;\mathrm{areas}}{\mathrm{Cross}\;\mathrm{sectional}\;\mathrm{area}\;\mathrm{of}\;\mathrm{the}\;\mathrm{pipe}}$$

With the increase of the number of holes, the diameter of holes gets smaller and the ratio of the total perimeter of holes to the total flow area of holes ‘α’ gets higher. On the contrary, ‘β’ decreases with the number of holes and is linearly related to the cavitation number. As ‘β’ decreases, the cavitation number decreases too. In the treatment of both the aqueous phase and organic phase, the cavitational intensity tends to be strongly affected by the ratio of the total perimeter of holes to the total flow area ‘α’ [43]. The **α** can be increased by changing the shape of the throat from circular to rectangular, increasing the number of holes (orifice), or changing the ratio of the throat/ hole perimeter.

## 3 Hydrodynamic cavitation-based processes for the treatment and processing of organic liquids

**Fig. 2:**
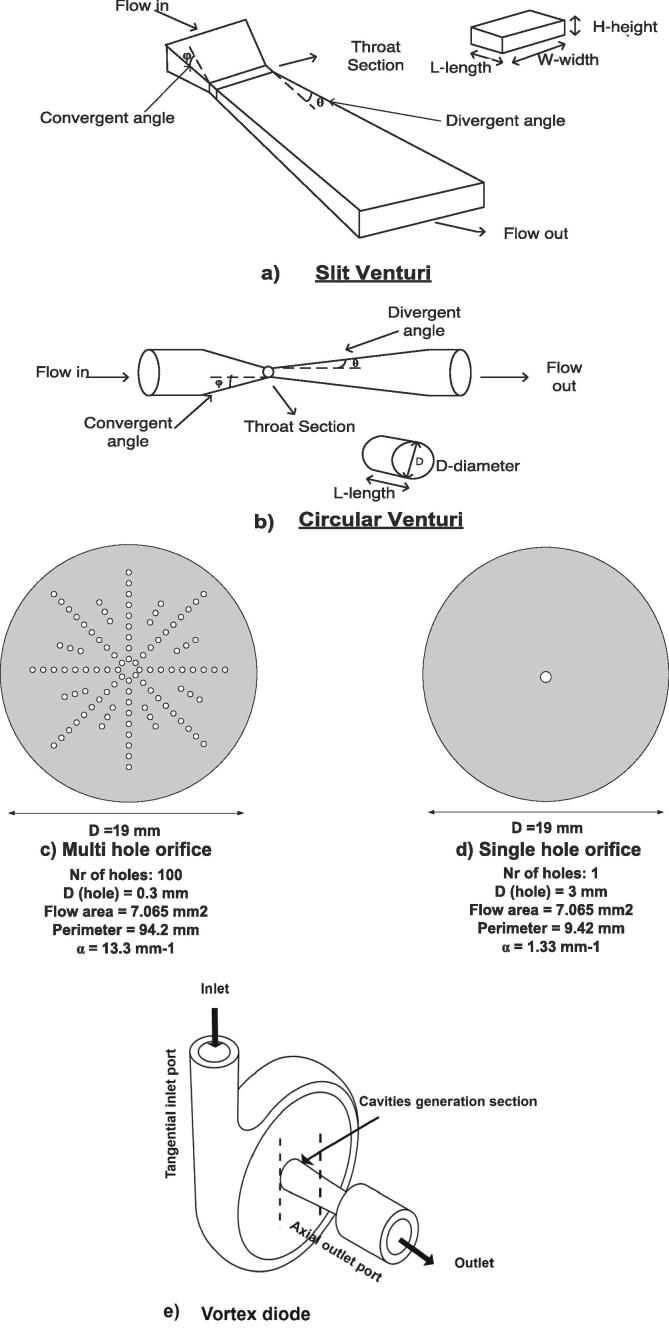
Different types of hydrodynamic cavitation devices used for the treatment in the organic phase; a) slit Venturi b) circular Venturi [[41], [57]]; c) single hole orifice d) multi-hole orifice [42]; e) vortex diode [40].

The passage of liquid through a constriction, including an orifice, Venturi, or vortex diode, causes a static pressure drop of the liquid, which consists of being below the threshold pressure for the cavitation, turning out in the generation of enormous amounts of cavities [44]. Under extreme conditions, molecules present in the implosion zone will dissociate into high oxidation potential reactive radicals. Relevant reactive radicals, including hydroxyl radicals (HO^•^) as well as H^•^, O^•^, HO_2_^•^, can be formed from the dissociation of the aqueous phase presented in the form of liquid oxidants into the organic phase [40]. Along with discussed significant factors, the type of used reactor affects the overall effectiveness of HC [45]. Different HC systems have been utilized to increase cavitation events, reactions yield and decrease the cost of treatment for HC-based processes. For example, Fig. 2 illustrates the most representative HC devices used for the treatment in the organic phase. The different HC reactors are commonly applied for the treatment of the organic phase are given below;

**(i) Orifice plate setup (single hole orifice and multi holes orifice):** The orifice is the next cavitation device reported in the treatment of the organic phase, as depicted in Fig. 2 **(c,d)**. Orifice thickness, defined as l/d (ratio of length to diameter l/d), is an important parameter that influences the intense collapsing of cavities. Theoretically, if the l/d ratio is below 2, it suggests that higher pressure rates and flow rates are required for the cavitation to occur, while if such a ratio is above 2, the pressure recovery rate can be controlled by increasing the orifice thickness [46]. The number of generated cavities increases with the number and diameter of holes [25]. The cross-sectional flow area is controlled by the number and diameter of holes [47].

**(ii) Venturi cavitation devices (circular and slit Venturi):** Venturi is a pipe constriction where cavities get formed in the throat section due to the decrease of static pressure below vapour pressure of the liquid [45]. As liquid flows through the downstream section, the cavities will collapse and implode, and thus release a vast amount of energy breaking the chemical bonds of pollutant molecules entrapped inside the cavity. The diameter of the throat, length of the throat, and divergence angle are important geometric parameters in controlling the cavitation intensity [48]. Among the applications of Venturi in wastewater treatment, the treatment of the organic phase, particularly vegetable oils, has also been performed revealing significant (%) yields of biodiesel [42]. From studies that include wastewater treatment, different types of Venturi, such as standard circular Venturi, slit Venturi, annular ring Venturi, annular slit Venturi and double annular slit Venturi, were compared to each other. Interestingly, it was found that using annular slit Venturi and double annular slit Venturi generated more cavities for given energy input [49]. Therefore, the use of slit and circular Venturi was encountered for the organic phase treatment, notably in biodiesel production **(**Table 1**; Nr 2,3),** Fig. 2 **(a, b)**. Higher biodiesel yield is usually obtained when the ‘α’ value is higher than 1.33 mm^−1^ and small cavitation numbers ranged from 0.19 to 0.34 (see Table 1). Higher biodiesel yield (90 %) can be achieved using a Venturi slit with an ‘α’ of 2.71 mm^−1^ compared to circular Venturi (82 %) which has a value of ‘α’ 2.0 mm^−1^ [27] **(**Table 1**; Nr 2, 3)**. Surprisingly, multi-hole orifices (100 holes) proved to give 99 % biodiesel yield, while an 87 % biodiesel yield was obtained for a single hole orifice in 5 min treatment time **(**Table 1**; Nr 5, 7)** [42]. It can be seen that the increase in the number of holes, from 8 to 24, increases ‘α’ from 4.00 to 8.89, ‘β’ decreases from 0.047 to 0.028 and the cavitation number values vary from 0.19 to 0.70 **(**Table 1**; Nr 8**–**11)** [29]. In the treatment of both the aqueous phase and organic phase, the cavitational intensity tends to be strongly affected by the ratio of the total perimeter of holes to the total flow area ‘α’ [43]. The **α** can be increased by changing the shape of the throat from circular to rectangular, increasing the number of holes (orifice), or changing the ratio of the throat/ hole perimeter.

**Table 1:** Dependence of biodiesel and desulfurization yield on the geometrical parameters of hydrodynamic cavitation devices.

Nr	Cavitation device	Shape	d (throat mm)	Flow area (mm^2^)	α (mm^−1^)	β	Cv	V (m/s)	(%) yield	Ref
**1**	Venturi	tube	1.4	–	–	–	0.29 (4 bar)	24.8	56.01 % (58.33 min) (biodiesel)	[44]
**2**	Venturi	slit	W-3.7 mm H-0.92 mm L-0.92 mm	–	2.71	–	0.33 (3 bar) (assumed)	–	90 % (60 min) (Biodiesel)	[42]
**3**	Venturi	circular	2	–	2.00	–	greater than0.33 (3 bar) (assumed)	–	82 % (60 min) (Biodiesel)	[42]
**4**	Venturi	Circular	3	7.065	1.33	–	0.30 (7 bar)	25.38	73 % (5 min) (Biodiesel)	[58]
**5**	Orifice plate	Single hole	3	7.065	1.33	–	0.49 (7 bar)	20.05	87 % (5 min) (Biodiesel)	
**6**	Orifice plate	Rectangular slit	L- 4.41 H- 0.50	7.065	4.42	–	0.46 (7 bar)	20.00	76 % (5 min) (Biodiesel)	
**7**	Orifice plate	100 holes	0.3	7.065	13.3	–	0.34 (7 bar)	24.18	99 % (5 min) (Biodiesel)	
**8**	Orifice plate	8 holes	1.00	–	4.00	0.047	0.7	11–40	≈ 15 % (80 min) (Biodiesel)	[43]
**9**	Orifice plate	12 holes	0.80	–	5.00	0.045	0.5	11–40	≈ 20 % (80 min) (Biodiesel)	
**10**	Orifice plate	16 holes	0.60	–	6.67	0.034	0.3	11–40	≈ 30 % (80 min) (Biodiesel)	
**11**	Orifice plate	24 holes	0.45	–	8.89	0.028	0.19	31	≈ 50 % (80 min) (Biodiesel)	
**12**	Orifice plate	Single hole	4.00	12.6	4.00	0.13	1.55 (assumed)	–	95 % (29 min) (Desulfurization)	[59]
**13**	Orifice plate	Single hole	1.00	–	–	–	0.15	–	17.9 % viscosity reduction (15 min)	[55]
**14**	Vortex diode	Circular throat	–	–	–	–	1.01 (2 bar; octanol) (assumed)	–	100 % (120 min) (Desulfurization)	[11]

**(iii) Vortex diode:** The use of a vortex diode, as a cavitation device represented in Fig. 2**e**, has been reported for several treatment processes, including desulfurization of fuels [11]. The principle of this device consists in increasing the tangential velocity which causes a pressure drop in the center of the vortex chamber; potentially, such pressure will further decrease as the fluid travels through the axial port towards the outlet port of the chamber [50]. Changing the size of the vortex chamber and vortex throat diameter affects the tangential velocity. When the length of the vortex chamber increases, from 0.6 mm to 1 mm, the velocity of heavy oil decreases from 49.1 to 36.1 m/s [30]. As the diameter of the vortex throat increases the cavitation decreases [51]. Unfortunately, no study defines the role of vortex throat diameter in the treatment of the organic phase. In these conditions, cavitation can be formed without the need for high pressures at the inlet of the cavitation chamber. Experimentally, high desulfurization rates using vortex diode are related to the large local pressure in the vortex core that is created from the vortex flow of fluid, which travels from low-pressure regions (p_v_) to high-pressure regions (up to 20 × p_v_) [51]. In contrast to linear flow cavitation devices, such as orifice and Venturi, swirling flow cavitating devices (e.g., vortex diode), proved to have early cavitation inception that starts at a pressure drop below 100 kPa [52]. Throughout the research, it has been found that in the case of linear flow cavitation devices, such as orifice and Venturi, cavitation takes place close to the device walls, causing the erosion of the device and lowering the device’s effectiveness over time, meanwhile in swirling flows devices, the cavitation takes place away from the walls of the device [53].

**(iv)High-speed homogenizers (mixers and rotating or spinning tube reactor):** Another type of HC reactor, such as high-speed homogenizer, was proved to be effective in the transesterification process with 97 % biodiesel yield [54]. Importantly, the geometry of the device stands out as a fundamental parameter in such applications; for example, the shape of the stator and the rotor part affects the geometry of the pathway for the processed liquid. High-speed homogenizers are less preferred devices at an industrial scale due to some disadvantages in terms of the high cost of treatment, high power consumption, and low control of cavitational processes [21].

Cavitation nozzle and vortex heat are the relevant devices used for the viscosity reduction of heavy oils utilizing HC [[55], [56]]. The principle of vortex heat generators (VTG) is to increase the diffusion of oil in a paraffin bubble, and the released energy at the implosion of cavitation bubbles is used to break chemical bonds of hydrocarbon compounds [56]. So far, there is scarce literature investigating the mechanism of these two cavitation devices in the treatment of the organic phase, and therefore, there is insufficient data to describe the process effectiveness utilizing pipe cavitation nozzle and vortex heat generators.

The following sections describe the process and mechanism involved in HC for different applications of organic phases, including desulfurization of fuels, heavy oil upgrade, and biodiesel production.

### 3.1 Enhancement of fuel desulfurization through hydrodynamic cavitation

Oxidative desulfurization is the most prominent method to reduce the sulphur content from most of the fuels due to the mild operating conditions. The sulphur in the fuel could be easily converted to separable forms, such as sulfones and sulfoxides. The sulfur compounds present in the various fuel fractions are sulphides, disulfides, mercaptans to refractory compounds, such as thiophene (T), benzothiophene (BT), dibenzothiophene (DBT) and such alkylated derivatives of thiophene. Therefore, much effort has been done into the desulfurization of thiophene derivatives. The investigation made by researchers is summarised in the supporting information (Table S1). The desulfurization of fuels using HC devices has been mainly performed by orifices and vortex diode. Promisingly, processes using vortex diode offered the highest efficiency (95 %) for the desulfurization of thiophene [40]. Due to the strong swirling flow in the vortex body, the cavities will move from the low-pressure region to the high-pressure region [51]. Cavity lifetime was estimated to be 2 × 10^-4^ s and the distance needed for a cavity to travel from inception to collapse was 1–2 mm, which become advantageous features in terms of early cavitation inception and higher cavitation yields [50]. Since the bubbles are found to be highly concentrated in the core of the vortex diode, the available contact area between cavities and organic phase droplets is increased and it turns out in high process effectiveness. For both instruments (vortex diode and orifice) at least 95 % desulfurization is still possible to be obtained [59]. Various parameters, such as pressure drop, concentration of initial S-compound, solvent phase ratio and nature of the solvents, were reported by Suryawanshi et al. The highest sulfur removal of 90 % was reported at the pressure drop of 2 bar and 5 bar for 2.5 % organic volume with octanol as the solvent [[29], [40]].

**Fig. 3:**
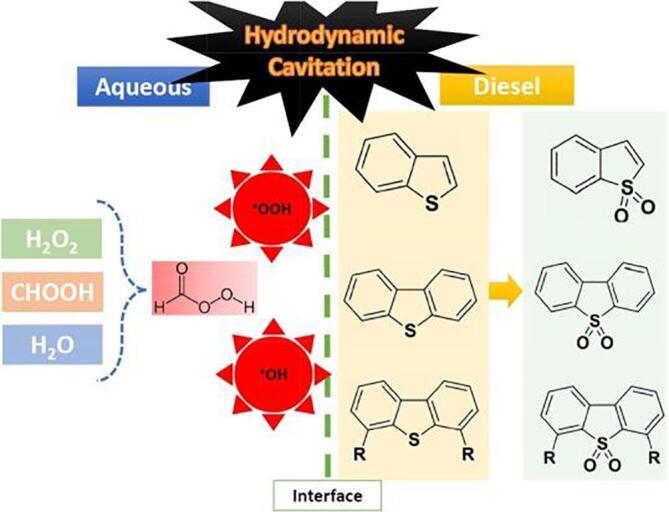
Mechanism for the desulfurization in HC– H_2_O_2_/formic acid system [59].

The control parameters, including the molar ratio of formic acid to oxidant, the molar ratio of catalyst to S-compounds, temperature, solvents and pH media, also require to be optimized to achieve higher (%) desulfurization yields of kerosene. It should be noted that an oxidant is mainly introduced as an aqueous solution, and thus a biphasic system (i.e., fuel-oxidant solution) is obtained. In the case of the desulfurization of kerosene utilizing HC, hydrogen peroxide (H_2_O_2_) and formic acid were used as oxidant and acid catalyst, respectively. The addition of H_2_O_2_/formic acid in the fuel under HC treatment assisted the oxidation of sulphur-based compounds contained in the organic phase. An initial S removal of 60 % was reported in the case of the sole HC process. But, when the oxidant H_2_O_2_/formic acid is introduced into the system a 90 % desulfurization was obtained. Increasing the molar ratio of formic acid to oxidant can indeed generate the number of hydroxyl radicals (^•^OH) and hydroperoxyl radicals (^•^OOH) via pyrolysis of hydrogen peroxide and water during cavitation collapse (Eqn 4-8), and the generated radical species in the aqueous phase will pass through the interfacial area to attack the sulphur-based compounds and concurrently oxidize them into sulfoxides and sulfones, and even more to SO_2_ and H_2_O, as expressed in **Eq. (**10–12) [60]. The mechanism of desulfurization in the HC– H_2_O_2_/formic acid system is given in Fig. 3.

(4) $$H_{2}O_{2}\overset{\mathrm{HC}}{\to }{\;}^{\cdot }OH\;+\;{\;}^{\cdot }OH$$

(5) $$H_{2}O_{2}+{\;}^{\cdot }OH\overset{\mathrm{HC}}{\to }{\;}^{\cdot }OOH\;+\;H_{2}O$$

(6) $$H_{2}O\overset{\mathrm{HC}}{\to }{\;}^{\cdot }OH+{\;}^{\cdot }H$$

(7) $${\;}^{\cdot }OOH\;+{\;}^{\cdot }OOH\overset{\mathrm{HC}}{\to }H_{2}O_{2}+\;O_{2}$$

(8) $${\;}^{\cdot }OH\;+{\;}^{∙}OH\overset{\mathrm{HC}}{\to }H_{2}O+\;O_{2}$$

Increasing the formic acid/oxidant molar ratio leads to an increase of (%) desulfurization, due to the formation of peroxyformic acid, this latter acid will further decompose into formyl (^•^CHO) and hydroperoxyl radicals (^•^OOH) that aim to attack sulphur-based compounds while the remaining amount will reform formic acid **(see Eq. (9**–**13))** [60].

(9) $${\text{HCOOH + H}}_{2}{\text{O}}_{2}\overset{\mathrm{HC}}{\to }{\;\text{CHOOOH + H}}_{2}\text{O}$$

(10) $$\mathrm{CHOOOH}\overset{\mathrm{HC}}{\to }{\;}^{\cdot }CHO\;+{\;}^{\cdot }OOH$$

(11) $$\mathrm{Th};\;BT;\;DBT\;\left(alkylated\;derivatives\right)\;+\;{\;}^{\cdot }OOH\;\overset{\mathrm{HC}}{\to }\;Sulfoxide\;+{\;}^{\cdot }OH$$

(12) $$\mathrm{Sulfoxide}\;+\;{\;}^{\cdot }OOH\;\overset{\mathrm{HC}}{\to }\;Sulfone\;+\;{\;}^{\cdot }OH$$

(13) $${\;}^{\cdot }CHO\;+\;{\;}^{\cdot }OOH\overset{\mathrm{HC}}{\to }\;HCOOH$$

If the molar ratio of acid/oxidant is increased beyond the optimum value, an adverse effect will occur, and (%) desulfurization will decrease due to the decarboxylation of formic acid at the bubble liquid interface, as described by **Eq.** (13), (14), [59].

(14) $$\mathrm{HCOOH}\;+\;{\;}^{\cdot }OH\;\overset{\mathrm{HC}}{\to }\;{\;}^{\cdot }CHOO\;+\;H_{2}O$$

(15) $${\;}^{\cdot }CHOO\;+{\;}^{\cdot }OH\;\overset{\mathrm{HC}}{\to }\;CO_{2}\;+\;H_{2}O$$

The (%) desulfurization of thiophene derivatives using HC has been reported only in the presence of an oxidation system with hydrogen peroxide/ formic acid, meanwhile, the application of acetic acid was reported within the desulfurization of fuels aided by acoustic cavitation. Thanks to the disadvantages of formic acid (such as instability, easy decomposition at ambient and higher temperatures and carcinogenic character), acetic acid is highly recommended to be used in desulfurization processes assisted by cavitation [61]. The processes with the above oxidants led to the removal of 65 %, 96 % and 97 % Sulfur from the different compounds, such as BT, DBT, and 4,6-Dimethyl dibenzothiophene (DMDBT), respectively. Other oxidants used along with H_2_O_2_ in these processes are peracetic acid and Phosphotungstic acid. The formation reactions of reactive species using these oxidants are given in equations 10–15. These catalysts were effective in the S removal, where 99 % of the S removal was reported. In addition, Fenton reagents, TiO_2_, and TiO_2_/H_2_O_2_ system in combination with ultrasound resulted in 80 %, 80 %, and 100 % S removal from dimethyl disulphide, respectively. Another way to enhance the desulfurization processes by cavitation is the addition of a phase transfer agent (PTA). The common PTA are quaternary ammonium salts which would be able to form complexes with the oxidant in the aqueous phase and drag them to the organic phase, where it can enhance the oxidative desulfurization. A study conducted by Bhasarkar et al., found that the DBT reduction was increased to 30 % and it can be further improved with the increase in PTA concentration (tetraoctylammonium bromide, TOAB) in US/Performic acid system [62]. Mei et al. reported a 98 % reduction of DBT using US/H_2_O_2_/ Phosphotungstic acid with the inclusion of TOAB as the PTA, while for the BT was 88 % [63]. All these studies opened up the possibility of combining these oxidants with the HC process as well.

In the oxidant system, the low pH favours the formation of peroxyformic acid, and peracetic acid. These possess a strong oxidising character than their precursor species. In addition, it is well known that the Fenton processes were effective in the generation of reactive species only at the acidic pH. In short, since the acidic pH media favours the desulfurization of fuels, the pH of the aqueous phase (acid/oxidant) should be adjusted to approximately 2 before introducing it into the reaction system [64].

Another factor that influences the overall rate of desulfurization is the initial concentration of sulphur compounds. For a range of 100–300 ppm S-containing model fuel, the highest desulfurization was achieved for the treatment of 300 ppm model fuel, and it can be explained by the increased probability of finding S-compounds for desulfurization. Thus, cavitation-based processes can be surely used as methods for the pre-treatment of fuels, followed by final-deep desulfurization by hydrogenation-based processes. In summary, it can be appointed that, HC processes are efficient in the desulfurization of fuels. The most effective HC systems are vortex diode and orifice. Desulfurization is favoured to take place under acidic pH media (ca. pH 2). Also, aqueous oxidants, such as performic and peracetic acid, can yield higher desulfurization reaching even 90 %.

#### 3.1.1 Oxidation mechanisms of S-compounds and extraction techniques toward oxidized S-compounds

As given in the previous sections, the desulfurization in cavitation is caused by the pyrolytic effect, and reactive species (^●^OH, and ^●^H). Furthermore, the cavitation processes enhance the mass transfer of the S- compound by enhancing the micro-mixing and emulsification. Extreme conditions of cavitation will break the sulphur bond and be oxidized by radicals, resulting in the formation of sulfoxides and sulfones. Similar to other radical-mediated degradation, desulfurization depends on the reactivity of the radical species towards the sulphur group. The reactivity further depends directly on the electron density around the S atom. The ^●^OH attacks at the electron-rich S centre to form sulfoxides and sulfones. The reactivity of sulphur species in the fuel is related to the property of high electron density, which means that S-compounds attached with electron-rich aromatic rings are more favourable to get faster oxidized through the electrophilic addition mechanism; furthermore, the presence of alkyl groups increases the electron density in the molecules [65]. Chen et al. studied the correlation of sulfides to sulfone conversion with different organic sulfur compounds. The electron densities on the S atom are 5.696, 5.739, 5.758 and 5.760 for T, BT, DBT, and 4,6- DMDBT, respectively. The order of reactivity is defined as follows 4,6-DMDBT> DBT > BT > T [[11], [66]]. Based on density differences, at the end of treatment, aqueous oxidant will separate from liquid hydrocarbons, holding with them oxidized S-compounds (sulfoxides and sulfones), which are continuously extracted through the water phase. This assures a partial decrease of the total content of sulphur from the fuel. Moreover, in the case of disulfides in comparison to thiols and sulfides much higher rate constant values were reported in the literature for reaction with hydroxyl radicals. It follows a different mechanism, as in the case of disulfides hydroxyl radicals tend to attack directly the weak S—S bond. In the case of sulphides, a slower two main pathways can take place – formation of S--–OH adduct or H-abstraction on secondary alkanes [[67], [68]]. In the case of thiols, these compounds under oxidative conditions can undergo conversion to disulphides. Such effects can overlay on the main reaction pathway, making the results difficult for detailed analysis, when trying to provide some general rules of sulfur compounds’ degradability under studied conditions. In comparison with other oxidative desulfurization processes, the cavitation phenomenon is associated with luminescence. This high energy emission is also capable of generating additional radical species through the decomposition of oxidants.

Based on the high polarity of sulfoxides and sulfones, they can be extracted from the organic phase using polar solvents through a liquid–liquid extraction technique or via adsorption. The right solvent must be selected considering specific parameters, such as solvent capacity, (which means that the solvent should dissolve only sulfones from treated fuel), and reusability of solvent (considering the low solubility of hydrocarbon mixture in the solvent, solvent/oil sample ratio, and the number of extraction stages) [4]. At this point, acetonitrile, methanol, *N, N*-dimethylformamide (DMF), butyrolactone, or *N, N*-dimethyl pyrrolidone represent the most common solvents used for extraction [[69], [70]].

The most common method for extracting sulfones from the organic phase is washing the samples after treatment with a suitable ratio of solvent/oil, and the extraction solvent can be introduced directly into the organic phase with the addition of oxidants [[71], [72], [73]]. Almost complete desulfurization of 99 % was achieved after ultrasonic cavitation treatment, followed by extraction of sulfones using acetonitrile at 1:1 v/v ratio and then centrifugated at 3000 rpm to completely separate the phases [74]. Using methanol as an extracting solvent in the ratio of 4:5 (v/v) solvent/oil, 90 % sulphur removal was obtained after HC treatment, followed by manual extraction [75]. During the desulfurization process using HC, methanol was directly introduced in the organic phase with oxidizing agents, obtaining a 99 % desulfurization [76]. Alternatively, adsorptive removal of sulfones after cavitational treatment can be performed using several materials, such as alumina oxides, activated carbon, zeolites, and polymers [70].

### 3.2 Heavy oil upgrading using hydrodynamic cavitation

It is well known that the heavy oils and vacuum residue are determined by characteristic parameters, such as density (kg/m^3^), viscosity (Pa × s), pour point (°C), carbon residue (wt%), and group composition (wt%) (paraffin, naphthene, aromatics, resins, asphaltenes), which after cavitation tend to reach lower values [77]. For this reason, cavitation represents a promising method in the heavy oil upgrading industry in terms of cost-effectiveness and (%) viscosity reduction in comparison with other conventional methods, such as carbon rejection, hydrogen addition processes, and separation processes [15]. Cavitation also helps the production of low molecular weight with the limited formation of unwanted coke. Two routes for heavy oil upgrade consist of the formation of free radicals from molecules of lighter organic and inorganic compounds, which serve as hydrogen donors and the thermal cracking of molecules in crude oil [[27], [78]]. The thermal cracking process leads to the formation of lighter molecules since C—C bond breakage occurs, and the highly energetic free radicals enhance the process thanks to the breaking of the carbon chain. Hydrogen radicals produced by thermal cleavage of O/C—H, in the presence of hydrogen donors, including water, tetrahydronaphthalene, and hydrocarbons (such as naphtha, pentane, light crudes), can generate the required hydrogen for hydrogenation of heavy cuts [[79], [80], [81]]. The presence of hydrogen donors converts heavy molecules into lighter ones and thus decreases the viscosity of heavy oils. The reaction pathway of heavy oil upgrading under sonocavitation treatment can be described by the so-called Rice mechanism, this latter mechanism involves three groups of reactions, such as (i) free radical generation (Eqn 16–19), (ii) propagation (Eqn 20–22), and (iii) termination (Eqn 23–28) [27], as described:

(16) $$RR^{\prime }\overset{)))}{\to }\;R^{∙}\;+\;R^{{∙}^{\prime }}$$

(17) $${\text{H}}_{2}\text{O}\;\overset{)))}{\to }{\;\text{H}}^{∙}{\;\text{+ HO}}^{∙}$$

(18) $${\text{H}}_{2}\;\overset{)))}{\to }{\;\text{2H}}^{∙}$$

(19) $${\text{RH + HO}}^{∙}\overset{)))}{\to }{\;\text{H}}_{2}{\text{O + R}}^{∙}$$

(20) $$R^{∙}+\;R^{\prime \prime }H^{∙}\;\overset{)))}{\to }\;RR^{∙}\;+\;H^{∙}$$

(21) $$H^{∙}+\;R^{\prime \prime }H\;\overset{)))}{\to }\;HR^{\prime \prime }H^{∙}$$

(22) $$HO^{∙}+\;R^{\prime \prime }H\;\overset{)))}{\to }\;HOR^{\prime \prime }H^{∙}$$

(23) $$R^{∙}\;+\;R^{{∙}^{\prime }}\overset{)))}{\to }\;RR^{\prime }$$

(24) $${\text{R}}^{∙}{\;\text{+ H}}^{∙}\overset{)))}{\to }\;\text{RH}$$

(25) $${\text{R}}^{∙}{\;\text{+ HO}}^{∙}\overset{)))}{\to }\;\text{ROH}$$

(26) $${\text{H}}^{∙}{\;\text{+ HO}}^{∙}\overset{)))}{\to }{\;\text{H}}_{2}\text{O}$$

(27) $${\text{H}}^{∙}{\;\text{+ H}}^{∙}\overset{)))}{\to }{\;\text{H}}_{2}$$

(28) $${\text{HO}}^{∙}{\;\text{+ HO}}^{∙}\overset{)))}{\to }{\;\text{H}}_{2}{\text{O}}_{2}$$

The different advantages of using cavitation technologies in the heavy oil upgrade include: a) the heating temperature of viscous oil will be increased by 2–3 °C without applying an extreme temperature source, b) less fuel consumption compared with conventional treatment methods, c) improvement of rheological properties, d) higher yields of light cuts [30]. To date, the different HC reactors are based on orifice, cavitation jet/ cavitation nozzle, vortex heat generator, and vortex diode (VTG). This latter concept, as a cavitation device, has shown good effectiveness in the treatment of heavy oil. For instance, it has been observed that the viscosity of heavy oil increases in the first minutes of cavitation treatment (C_v_ 0.08) and then it decreases until the end of the process; however, the addition of the right hydrogen donors (e.g., gasoline) contributes to reducing the viscosity of heavy oil by almost 19 % in 15 min [30]. The utilization of the orifice as a cavitation device (C_v_ 0.15) resulted in the viscosity decrease of VR (vacuum residue) + kerosene by 17.9 % in 60 min treatment time [78]. The presence of lighter fractions of crude oil and volatiles in vacuum residue assists the scission of carbon bonds in alkanes, aromatics, paraffin, naphthene, while olefins were assisted by the thermal cracking [78]. Another type of cavitation device, such as cavitation nozzle, was also demonstrated to be effective in the treatment of heavy oil. Cavitation aided by hydrogen donors, e.g., tetrahydronaphthalene (THN), resulted in increasing the viscosity reduction rate by 15.7 % [55]. In another way, the water, as a H_2_ donor, changed the viscosity of the heavy oil by 63 % compared to that of the bare medium, where the viscosity change was only 8 %. In addition, the combination of HC with microwave irradiation resulted in an increase in distillate yield from 1 to 3 % with activated carbon as an absorber of the microwave. The water medium also favours a good cavitation condition. The incorporation of iron nanoparticles in the cavitation process improves the upgrading of heavy oils by facilitating the hydrogenation reaction. A comparison of heavy oil upgrading studies through HC is given in Supplementary material (see **Table S1**). By analysing the excellent properties of oils obtained by HC processing, it can be concluded that: HC can be successfully employed for the upgrading of heavy oil, leading to a significant lowering of the viscosity of treated streams and more value-added products under ambient conditions. The addition of hydrogen donors to the system is crucial to avoid severe radical recombination leading to an increase in viscosity. Gasoline range distillates are suitable and relatively inexpensive additives that can be used as hydrogen donors in the hydrocarbon upgrading process (HCUP). A combination of vortex diode, and gasoline as a hydrogen donor, proved to be the most effective method compared with the orifice in the treatment of vacuum residue and a combined approach of cavitation nozzle and tetrahydronaphthalene (THN) as a hydrogen donor in the treatment of heavy oil.

### 3.3 Biodiesel production through hydrodynamic cavitation system assisted by catalytic and non-catalytic methods

Biodiesel production is based on the transesterification of triglycerides or the esterification of free fatty acids with higher-order alcohol, such as methanol or ethanol. Micro and macroalgae, animal fat and vegetable oil, food crops, lignocellulose material, among others, represent the major precursor for biodiesel production. The process typically involves the step-wise formation of glycerol from triglycerides. In order to achieve the maximum yield of biodiesel, the alcohol to oil molar ratio should be maintained at a minimum of 3:1 and the biodiesel is formed as the top layer [[82], [83]]. The catalysts used a production are basic, acidic, and lipase type. The alcoholises can be done by a variety of techniques, such as mechanical stirring, microwave heating, and supercritical methanol. However, the scaling up of the above techniques in biodiesel production is difficult to install at industrial level. Over the last decades, cavitation technologies combined with catalytic and non-catalytic methods were introduced as an alternative to enhance the biodiesel production yield. Edible or non-edible vegetable oils and animal fats are used in biodiesel production. In HC-assisted transesterification processes, the micro-fine bubbles affect the alcohol oil interface with the formation of supersonic alcohol jets. This speeds up the transesterification processes, and in most cases, more than 90 % of the conversion was reported within a short span of time. The catalysts used in these processes were NaOH, and KOH. The reaction mechanism of fatty acids methyl esters is described in Eq. (29–31) [84]:

(29) $$\text{Tryglycerides + ROH}\overset{\mathrm{catalyst}(NaOH;\;\text{KOH)}}{\to }\;\text{Diglycerides + FAME}$$

(30) $$\text{Diglycerides + ROH}\overset{\mathrm{catalyst}(NaOH;KOH)}{\to }\;\text{Monoglycerides + FAME}$$

(31) $$\text{Monoglycerides + ROH}\overset{\mathrm{catalyst}(NaOH;KOH)}{\to }\;\text{Glycerol + FAME}$$

The effectiveness of biodiesel production processes utilizing HC technologies depends on several factors including the type of reactor, oil to alcohol molar ratio, type and dosage of catalysts, temperature, and reaction time [85]. When compared with all cavitation devices for the conversion of vegetable oils to biodiesel, multi-hole orifice (100 holes; 3 mm diameter) has been identified as the most effective device, offering 99 % biodiesel yield for 5 min treatment time [42]. In the case of Venturi-based cavitation devices, the effectiveness in biodiesel yield follows the order: General type of Venturi (95.6 %; 8 min) > slit Venturi (90 %; 60 min)> Circular Venturi (82 %; 60 min) [42]. Higher transesterification yields could be achieved for the increased value of α (2.71 mm^−1^) for slit Venturi and (2.0 mm^−1^) for circular Venturi. The multi-hole orifice has exhibited higher biodiesel yields compared with Venturi due to the reduced turbulence intensity in the convergent angle of Venturi. The interaction of cavities will lead to the coalescence of bubbles and sphericity loss, which results in a decreased cavitation intensity in the Venturies [29]. The use of a rotating generator in an HC system resulted in the conversion of 99 % m/m FAME, which would be installed in a minimum space.

Higher biodiesel yield can be achieved for a higher number of holes in the orifice due to increased cavities generation spots. Higher biodiesel yield was obtained for 25 holes orifice with 95 % in 10 min treatment time, meanwhile using 24 holes orifice, 75 % biodiesel yield could be achieved in 40 min [[86], [87]]. The longest treatment time to achieve 90 % conversion yield in the transesterification processes was obtained using high-speed homogenizers with 120 min, while a single hole orifice obtained a similar conversion with 90 min treatment time [[54], [88]].

The next relevant parameter affecting the transesterification process is the molar ratio of oil to alcohol. In general, increasing the molar ratio up to an optimum value results in the enhancement of biodiesel production and the shortage of treatment time [89]. For example, the increase of the molar ratio, from 1:4 to 1:6, was conducted to an increase in biodiesel yield from 45.2 % to 98.1 %, respectively, in 15 min treatment time [28]. Apart from the molar ratio, the catalyst dosage also becomes relevant in such processes. Herein, increasing the catalyst dosage (e.g., H_2_SO_4_), from 1 to 2 wt%, decreased the treatment time from 110 to 90 min, respectively; meanwhile, the (%) biodiesel yield was slightly increased from 92 to 95 % [[88], [90]]. The common catalysts are sodium hydroxide (NaOH), potassium hydroxide KOH (in the range of 1–3 wt%), sulfuric acid H_2_SO_4_ (1, 2 and 10 wt%), potassium methoxide pellet, Na_2_CO_3_ (0.5–1.5 mol/L), Ca(OH)_2_ (0.5 mol/L) (**see** Supplementary material**; Table S1: Nr 8**–**14**). It is important to point out that increasing the catalyst dosage beyond an optimum value may result in a decrease in transesterification yield due to the soap formation, since the excess of NaOH or KOH reacts with free fatty acids in the reaction system. The selection of catalyst is also a critical step since such catalyst dictates the effectiveness of the process in terms of high biodiesel yield, short treatment time, low reaction temperature, and base catalysts, which are considered to fulfill all listed requirements; interestingly, acid catalysts are used when the oil has a consistency of high concentrated free fatty acids and water [91].

Besides improving biodiesel production, the ultrasonic system could be connected in parallel with the HC system. In this system, the reaction time was reduced by 30 times than the conventional processes. Here 92 % yield was reported in just 5 min [92].

#### 3.3.1 Characterisation of biodiesel properties synthesized through hydrodynamic cavitation-based processes

The HC can lower the values of kinematic viscosity, acid value, and flash point of the produced biodiesel compared with the conventional ultrasound and methods. According to the standard specifications (e.g., ASTM D6751), the value of a flash point should be ranged from 100 to 170 °C. The flash point of biodiesel samples using HC exhibited the lowest flash point of 151 °C compared with ultrasound (155 °C) and conventional methods (168 °C) [41]. Due to the lower mass transfer rate in the conventional method, a considerable amount of oil remains unreacted in the final product, thus dictating low conversion of oil into biodiesel [41]. Similar properties were obtained after biodiesel yield by means of HC using high throat perimeter to flow area ratios, where the flashpoint of biodiesel was evaluated to be 166 °C [42]. The acid value is another parameter that specifies the amount of free fatty acids, and for a higher amount of fatty acids, a bigger acid value can be obtained. The higher acid value indicates the risk of corrosion of the engine, therefore, the value should be lower than 0.50 mg KOH/g FAME as specified in ASTM D664 standard method [81]. In a recent development, Chuah et al. [28] reported an acid value of 0.30 mg KOH/g FAME, while the cetane number (CN) (approximately 60) was evaluated to be higher than the standard value (ca. 50) after HC. All reported results allow concluding that the use of HC in biodiesel production leads to the synthesis of high-quality biodiesel, thus meeting the established parameters in standard methods.

## 4 Applications of acoustic cavitation for organic phase processes

As mentioned previously, cavitation conditions can be generated through an ultrasonic probe (also known as sonotrode) or ultrasonic horn; alternatively, it is also possible in an ultrasonic bath equipped with ultrasound transducers, which are characterized by frequency and ultrasonic power. The ultrasonic power, transducer diameter, and operating temperatures, are important parameters to control the effectiveness of the process. The mass transfer rates of liquid can be increased by increasing the ultrasonic power, decreasing the distance between the sonocavitation reactor and ultrasonic irradiation source, decreasing the transducer diameter, and raising the temperature [93]. The transducers are classified into three different groups, including gas-driven (dog whistle; syren), liquid-driven (whistle type transducer), and electromechanical transducers (piezoelectric and magnetostrictive transducer) [94]. In studies about the treatment of organic phase employing cavitation, ultrasonic bath, electromechanical piezoelectric transducers and ultrasonic probe represent the most used equipment using a titanium probe tip or titanium probe horn. The cavitation intensity decreases with the increase of distance from the ultrasonic horn [95]. Except for the ultrasonic power and frequency, the cavitation activity can also be controlled by interesting factors such as flow regime (i.e., laminar or turbulent), the presence of solid particles in the liquid media, as well as the ratio of solid phase/liquid phase [96]. The ultrasonic irradiation surface affects the cavitation intensity in the liquid media, for example, a bigger ultrasonic surface contributes to the increased number of cavitation events. The use of an ultrasonic bath with a longitudinal transducer at the bottom will eventually generate a greater number of cavitation zones than a single ultrasonic horn [95]. Employing high ultrasonic frequency devices (170 kHz) increases the cavitation intensity due to the greater number of generated bubbles with a small diameter, meanwhile low-frequency sonoreactor (68 kHz) generates less bubbles with a bigger diameter, which is translated to decreased cavitation events [97]. On the other hand, the ultrasonic power affects the distribution of the generated bubbles, the initial radius of bubbles, and oscillations velocity, and with the increase of the ultrasonic power, all three factors increase linearly. Very recently, Sajjadi et al. [98] revealed that by increasing the power of the ultrasonic transducer, from 100 to 400 W, the radius of the bubble was 4.53 times greater than the initial radius, and the oscillation velocity increased from 8.76to 13.65 cm/s, and the distribution of cavities in the media was increased by 0.6 %. Importantly, all liquid will be uniformly distributed around the transducer since the acoustic cavitation streaming will push the liquid downwards and upwards unceasingly [98]. This feature becomes relevant when both organic and aqueous phases are mixed, and equal distribution of aqueous phase in the organic phase is needed to contribute to a greater number of generated bubbles in the system.

### 4.1 Desulfurization of fuels assisted by acoustic cavitation

The efficiency of acoustic cavitation in fuel desulfurization has been studied throughout the last years. To evaluate the effectiveness of these processes, important factors, such as frequency, power density (W/L), type of oxidants, and type of catalysts, should be carefully considered. A frequency range of 20–50 kHz is preferred in the ultrasound-based desulfurization process. The acoustic cavitation power and volume of processed fuel define the cavitational yield, being expressed as power density (W/L). For higher ultrasonic power (above 150 W) and small processed volumes (between 10 and 50 mL), the power density has exceeded 10 000 (W/L), which was differently translated to a desulfurization from 90 to 100 % in a short processing time of 15–30 min. Since the processed volume is relatively small, the whole media is intensively irradiated, and a consequently bigger number of cavities are generated. For instance, the biggest power density of 30 000 (W/L) was calculated using a 750 W ultrasonic probe in 25 mL fuel, where above 95 % desulfurization could be obtained [[76], [99]]. Decreasing ultrasonic power to 70 W for processing of 38- and 28-mL fuel lead to a decrease in cavitation yield, which correspond to 47.05 % and 32.56 % desulfurization, respectively, and the treatment time increased to 90 min [[62], [100]].

Higher desulfurization yields were also reported when low ultrasonic powers were introduced for the treatment of small volumes of fuels. In this case, increased desulfurization value was majorly attributed to the added effect of oxidants and catalysts present in the system. The oxidation system of the US- Fenton’s reagent is a successful and promising approach to achieving high desulfurization rates in a short treatment time [101]. In the presence of ferrous ions (Fe^2+^), hydrogen peroxide will be decomposed, and a higher number of hydroxyl radicals will be produced, while ferrous ions themselves will be oxidized to ferric ions (Fe^3+^). The role of ferric ions is to catalyse H_2_O_2_ and regenerate hydroxyl radicals until the amount of H_2_O_2_ is consumed [101]. The power density for this oxidation system was proved to be 333 (W/L) **(see** Table 2**; Nr 1)**.

**Table 2:** Effect of power density in the desulfurization of fuels.

Nr	Equipment	Frequency (kHz)	Power (W)	Volume (L)	Power density (W/ L)	(%) Desulfurization	Ref
**1**	Ultrasonic self-designed equipment	28	200	0.6	333	98.3 (15 min)	[101]
**2**	Ultrasonic bath	33	250	0.01	25 000	100 (15 min)	[102]
**3**	Ultrasonic horn	20	100	0.5	200	99 (60 min)	[74]
**4**	Ultrasonic generator	42	185	0.1	1 850	100 (30 min)	[103]
**5**	–	24	400	0.03	13 333.3	90 (17 min)	[75]
**6**	Ultrasonic probe	20	750	0.025	30 000	98.75 (9 min)	[76]
**7**	Ultrasonic probe	20	150	0.03	5 000	99.47 (180 min)	[104]
**8**	Titanium ultrasonic probe	20	600	0.05	12 000	99.4 (10 min)	[63]
**9**	Titanium ultrasonic probe	20	250	0.025	10 000	75.23 (30 min)	[63]
**10**	Ultrasonic reactor	70	100	–	–	65.28 (10 min)	[105]
**11**	Titanium ultrasonic probe	20	750	0.025	30 000	95 (5 min)	[99]
**12**	Ultrasonic bath	37	150	0.010	15 000	98 (15 min)	[106]
**13**	Ultrasonic horn	19.9; 21.1	80	0.025	3 200	95 (30 min)	[107]
**14**	Ultrasonic probe	–	300	0.1	3 000	100 (40 min)	[108]
**15**	Ultrasonic probe	20	600	0.042	14 285.7	98.8 (10 min)	[109]
**16**	Ultrasonic probe	20	300	0.05	6 000	98 (30 min)	[110]
**17**	Ultrasonic processor	20	70	0.07	1 000	88.35 (30 min)	[111]
**18**	Ultrasonic bath	35	70	0.028	2 500	47.05 (30 min)	[62]
**19**	Ultrasonic apparatus	20	200	0.036	5 555.5	98 (120 min)	[112]
**20**	Ultrasonic probe	20	240	0.025	9 600	35 (90 min)	[113]
**21**	Titanium ultrasonic probe	20	200	0.16	1 250	94.8 (30 min)	[114]
**22**	Ultrasonic bath	35	70	0.038	1 842.10	32.56 (90 min)	[100]
**23**	Ultrasonic probe	21	400	0.05	8 000	80.85 (7 min)	[115]
**24**	Ultrasonic bath	35	35	0.025	1 400	77.5; 77.6; 77.9 (90 min)	[116]
**25**	Titanium probe tip	20	120	0.03	4000	95 (300 min)	[117]
**26**	Ultrasonic horn	19.85–20.05	400	0.05	8000	85.6 (pyrrole); 90 % (indole) (240 min)	[118]

The addition of photocatalyst among the liquid oxidant can further enhance the effect of cavitation in the oxidation of S-compounds. Coupled oxidation system of UV, TiO_2_, H_2_O_2_ and sonocavitation revealed 99 % desulfurization in 60 min time, and the value of power density was found to be 200 (W/L) **(**Table 2**; Nr 3)** [74]. Various types of adsorbents, known as carbon-based, silica-based, metal-modified adsorbents and graphene-based adsorbents, have been reported to be used in the desulfurization of fuels by means of acoustic cavitation **(**Supplementary material**; Table S2: Nr 2, 4, 12, 14, 21)**. Carbon-based adsorbents, such as Shirasagi TAC (trade name for a commercial activated carbon) and CFP-450 (derived from Cassia fistula biomass at 450 °C) modified with single metals (Ni, Zn, Co, Cu) and double metals (Ni and Cu), successfully improved the sulphur removal up to 100 % in 15 min processing [102]. Total desulfurization was firstly attributed to the intense cavitation and microstreaming, which increases the interaction surface area between adsorbent and sulphur molecules, herein, the power density for this system was found to be 25,000 (W/L) **(**Table 2**; Nr 2)**. Initially, S-compounds can get oxidized to sulfones and then adsorbed by a metal-modified adsorbent. Another type of catalyst-adsorbent, such as graphene oxide GO-COOH, which is a highly acidic and modified catalyst using chloroacetic acid, could yield 95 % desulfurization in 300 min treatment time. In this type of catalyst, adsorption-oxidation occurs on its exfoliated surface, which increases the interface layer between S-compound and catalyst **(**Table 2**; Nr 25)**. After the experimentation, high desulfurization rates, low treatment cost, and environmentally friendly treatment conditions suggest that the use of cavitation aided by adsorption technology represents a competitive alternative [117]. Specifically, since they can be prepared from different materials that ensure high surface area and high mechanical strength; eventually, the chemical modification of the adsorbent's surface with positively charged metals may boost the sulphur removal due to the interaction with negatively charged sulphur fraction [102].

From the reviewed literature, it can be summed up that the studied frequency range of acoustic cavitation devices was found to be in the range of 20–42 kHz and ultrasonic power of 35–750 W. The highest desulfurization yields could be obtained when a power density higher than 10,000 (W/L) is employed. However, with the use of oxidants and catalyst high desulfurization efficiency was achieved with a low power density (W/L). Fenton’s reagent, hydrogen peroxide (H_2_O_2_), acetic acid, sodium persulfate, ozone, potassium superoxide, photocatalysts (TiO_2_), carbon-based adsorbents and catalysts, metal-modified adsorbents and graphene-based adsorbents cover a broad range of oxidants and adsorbents-catalysts used for the oxidative desulfurization assisted by acoustic cavitation studies. It is recommended, that more studies should be performed for higher frequencies to check if better effectiveness would be obtained. Some recent reports suggest such an approach [33].

### 4.2 Application of acoustic cavitation for upgrade of heavy oil

Acoustic cavitation is a timely and efficient technique to process heavy crude oil with the purpose of viscosity reduction and concurrently producing lighter components. Heavy oil upgrading via acoustic cavitation can also be aided by the presence of catalysts, hydrogen donors, or temperature increase. Acoustic cavitation assists in breaking the C—C bonds and thus yielding lighter hydrocarbon cuts. Heavy oil upgrading was mainly performed using a probe-based cavitation device, as documented in Table 2. The diameter of the probe somehow influences the upgrading process of heavy oil, e.g., bigger diameter of the probe creates intense microstreaming which will vigorously push the liquid equally in all directions around the probe, this latter phenomenon increases the cavitation zone and cavitation intensity. Kaushik et al. [119], for instance, referred that by using a 7 mm probe diameter, the physico-chemical properties of vacuum residue were lower than using a 3 mm diameter probe. Among the reviewed development works, the ranges of the used ultrasonic frequency did not exceed 40 kHz, meanwhile, the ultrasonic power varied from 200 to 2000 W. Since the sonocavitation equipment, treated volume and oxidants differ from different studies, it is tough to make a fair comparison between the processes and thus define the best cavitation assisted system, but an approach has been made to evaluate the effectiveness of systems by calculating the power density (W/L) of each system. It has been observed that nickel-based catalysts assisted by acoustic cavitation exhibited higher yields of lighter components and reduced the viscosity due to the ability to easily dissolve in crude oil matrix and ensure a bigger number of active sites [120]. A modified type of nickel-based catalyst, such as NiO-SiO_2_, was demonstrated to successfully reduce the viscosity of heavy crude oil by 50–60 % in 120 min [121]. The effectiveness of heavy oil upgrading was ascribed to the role of acoustic cavitation assisted by catalyst nanoparticles since the process was performed under high ultrasonic power of 400 W and room temperature (ca. 25 °C). The use of acoustic cavitation is more beneficial compared with conventional thermal cracking since acoustic cavitation can generate higher light fuel fractions and concurrently inhibits coke formation. Using acoustic cavitation for the processing of residual oil, Song et al. [31] reported that lighter fractions of diesel (ca. 55 %) and gasoline (ca. 26 %) were obtained. For the same process, power density (W/L) was calculated to be 833.3 W/L in 120 min processing time. To eventually increase the light fractions yield, an increase in ultrasonic power will lead to higher power density and therefore long-chain alkanes can be broken into short-chain alkanes, and the viscosity of heavy oil will be significantly reduced. When ultrasonic power was increased from 200 to 2000 kHz, power density increased to 8000 (W/L), leading to higher yields of light components such as diesel (61.1 %) and gasoline (28.9 %) [122]. Power density (ca. 7500 W/L) successfully assisted in 11 % nitrogen conversion and 5 % viscosity reduction of heavy oil [123]. To increase the interfacial area between heavy oil and catalysts or liquid oxidants, it is important to introduce surfactants into the system. Surfactants, known differently as phase transfer agents, enhance the mass transfer between the aqueous phase (oxidant) and the organic phase, and they can stabilize the emulsions in the system through the readjustment of their structure and thus assist in the micelles dissemination [119]. Therefore, the generated radicals can be displaced, from the aqueous phase to the organic phase, and placed in the micelle in which the collision probability and reaction rates are higher [27]. The addition of non-ionic surfactant reduced asphaltene content (by 48 %) of vacuum residue compared with asphaltene reduction in the absence of surfactant (ca. 40 %) [119]. The combined effect of acoustic cavitation with hydrogen donors is a promising technology for refining heavy oil residues, resulting in viscosity reduction and a higher synergistic effect for the combined process of ultrasound and hydrogen donor addition [77]. At cavitation conditions, the hydrogen donor generates hydrogen free radicals, which inhibit the formation of coke and enhance lighter cuts formation and viscosity reduction. Treatment of vacuum residue in the presence of tetralin, as hydrogen donor, yielded a higher amount of generated light hydrocarbons, along with a reduction of viscosity, density, and pour point of processed feedstock [77].

### 4.3 Application of acoustic cavitation for biodiesel production

Ultrasound-induced cavitation and the base-catalyzed integrated process are reported as successful methods for obtaining enhanced biodiesel yield. Importantly, biodiesel production is strongly dependent on available cavitation area, ultrasonic power, ultrasonic frequency, type and amount of catalyst, and alcohol to oil molar ratio (**Table S4 in** Supplementary material). For higher ultrasonic power, higher transesterification yields can be achieved due to an increased number of formed cavities; for example, increasing 10-fold ultrasonic power, from 120 to 1200 W generated between 98 and 99 % biodiesel yield and concurrently decreased the treatment time from 35 to 10–20 min [[124], [125]]. The effectiveness of catalysts in the system is closely related to the size of cavitation bubbles. More intensive cavitation causes a decrease in cavities size, which will improve the mass transfer at the surface of the catalyst [126]. Since acoustic cavitation efficiency can be enhanced using different catalysts, the nanosized scale catalysts are considered to further extend the biodiesel yield due to the high surface area of nano-catalysts that results in a significant interfacial area and greater transesterification yield. Kelarijani et al. [127], for instance, used an ultrasonic bath (37 kHz, 1 000 W) to treat Rapeseed oil utilizing distinct nanomagnetic catalysts, such as Li/Fe_3_O_4_ and Li/ZnO-Fe_3_O_4,_ obtaining a 99.8 % yield in 35 min.

The solubility of the alcohol in the organic phase determines the overall yield of biodiesel production. Experimentally, introducing separately ethanol and *n*-butanol in an ultrasonic bath system (37 kHz; 50 W) for conversion of oleic acid, resulted in higher biodiesel yield, e.g., the presence of *n*-butanol revealed a 98 % yield, which was slightly higher compared with ethanol (96 %), this was due to the fact that butanol presents higher solubility in oleic acid [126]. Another important feature to boost high biodiesel yields regards the selectivity of catalyst with basicity properties. Highly basic catalysts tend to exhibit higher biodiesel yield. When comparing three different heterogeneous catalysts (such as ZnO/Al_2_O_3_, MgO/Al_2_O_3,_ and SrO/Al_2_O_3_) for the transesterification of rapeseed oil, it was suggested that even lower loadings of SrO/Al_2_O_3_ (2 wt% SrO) offered an increased biodiesel yield of 97.46 % compared with higher loadings of ZnO/Al_2_O_3_, MgO/Al_2_O_3_ (5 wt%) thanks to its highly basic properties compared to two MgO and ZnO [128]. This fact becomes relevant from the economical point of view of synthesis costs related to such nanomaterials, the usage of small quantities of nanomaterials will obtain more economically profitable processes. The incorporation of enzymes in biodiesel production is recognized as an alternative to achieving successful results. Potentially, the types of enzymes can sometimes surpass the effect of ultrasonic power leading to the high efficiency of the reaction. As an example, introducing Thermomyces lanuginosus (Lipozyme TLIM) and Novozym 435 enzymes in an ultrasonic system (20 kHz, 120 W) and ultrasonic bath (40 kHz; 250 W) for the treatment of waste cooking oil (WCO) yielded around 96 % biodiesel over 180 min [[129], [130]]. Particularly, due to the intense collapse of the cavities, acoustic cavitation may affect the enzyme structure due to the breakage of the bond between the enzyme and supporting material, which leads to denaturation of the enzyme and therefore affects the overall reaction [[131], [132]]. In contrast to the effect of temperature on the denaturation of the enzyme, sonocavitation will not change the active sites of the enzyme [133]. Except for the treatment of oil to produce biodiesel, sonocavitation can also be applied in the extraction of oil from seeds [134]. The extraction utilizing cavitation can be explained by the physical effect of cavitation that occurs on the surface of seeds, increasing the penetration of solvent into the seed. Oil extraction yield increases with ultrasonic power and decreases when rising temperature, as it is lowering the cavitational effect [134].

In general, from all reviewed studies based on biofuel production utilizing acoustic cavitation, ultrasonic horn and ultrasonic bath stand as the promising processes in the treatment of different oils. In both cases, it was reported to achieve above 90 % transesterification yield, but treatment time and cost of the experiment differentiate them in terms of the most effective method for biodiesel production. Since the process conditions strongly differed, it is difficult to make a fair comparison among studies and thus define the best configuration of the system.

## 5 The role of cavitation in enhancement of mass transfer and reaction rate by ability to form emulsion system

Emulsification, identified as the process of mixing two immiscible phases, increases mass transfer in these applications as the interfacial area is highly increased by dispersing one liquid into the second one. Thus, it can form a beneficial environment for the effective performing of several reactions in multiphasic systems even when both phases remain non-miscible [135]. A simple stirring of the organic and aqueous phase is not efficient enough to form emulsions, in which cavitation has proved to be an effective method of nano-emulsions formation [136]. In short, the cavitation is possible to form an emulsion even at low power consumption. The emulsion formed as a result of cavitation is highly stable, which can further be increased using surfactants that reduce the interfacial tension among the phases and promotes the droplet breakup [[137], [138], [139]]. The formation of the emulsion is attributed to the physical effect of HC [59]. Similar to previous applications, the configuration of the cavitation device is highly important in the generation of the emulsions, where the throat area to pipe area ratio (β) lies as an important parameter to generate a nano-emulsion (e.g., high ratios are desired), while Venturi-based devices are found to be more beneficial compared with orifice plates [57]. The production of nano-emulsions, with droplet size smaller than 100 nm, was obtained using a HC device (cavitation number between 0.17 and 0.20) and in presence of surfactants, such as Tween 80 and Span 80 [57]. In this case, cavitation is able to induce high interaction between polar and non-polar phases, which results in the formation of the emulsion. Some reactions can take place directly on the interfacial area, which is highly increased when the emulsion is formed, and as a result, higher reaction efficiency can be achieved [140].

Cavitation also enhances the dispersion of organic and aqueous phases, and consequently, the emulsion formation increases the desulfurization rate by promoting heterogeneous reactions and improving the activity of the oxidants [111]. Margeta et al. have reported a 95.38 % sulphur removal from a model fuel (containing 3976.86 ppm DBT in 39 wt% *n*-heptane, 28.5 wt% *n*-dodecane and 30 wt% *n*-hexadecane) [111]. A similar effect of emulsion formation can be obtained using acoustic cavitation. Even though the emulsion formation enhances with the increase of irradiation time, there is a limitation of the droplet size, and further effectiveness remains dependent on treatment time only. However, it was observed that beyond the optimum time, no extent of the desulfurization can be observed [75]. Sinhmar and Gogate reported that the maximum desulfurization rate could be achieved in 120 min [141]. This outcome was credited to the remaining S-compounds that are not reactive enough to undergo the oxidation reaction at applied process conditions.

The process optimization for emulsion formation depends on the type of cavitation used. For example, in the case of acoustic cavitation, the increase of the ultrasonic power causes an increase in emulsion temperature, followed by a decrease in interfacial tension and viscosity which is superior for the formation of smaller droplets [142]. Regarding HC, the droplet size can be lowered by two distinct scenarios, *i)* increasing inlet pressure that consequently causes higher linear velocity and thus higher turbulence of the flow and cavitation intensity, and *ii)* the number of cavitation passes [143]. For instance, HC proved to offer 8-fold higher efficiency in a 6-fold shorter time reaction compared with mechanical stirring. This was due to the ability of HC to form smaller droplet size emulsion that increases the interfacial area of the oil/aqueous phase conducting to higher conversion rates [84]. Using a seven-hole orifice in HC was also a prevailing method in emulsification formation compared with mechanical stirring [144]. Here, sub-micron scale emulsions (ca. 476 nm) were produced using a liquid whistle HC rector (LWHCR) [[145], [146]].

Concerning oil in water emulsions, the implementation of HC allowed generating sub-micron 100 nm droplets with high stability (up to 8-months). Depending on the characteristics of the organic phase, the average droplet size strongly differed, e.g., 68 nm (heptane/water), 19 nm (castor oil/water), and 27 nm (soybean oil/water) droplets were obtained [147]. However, the addition of specific amphiphilic catalysts, such as polymerized metal alkoxide (titanium isopropoxide (Ti(Pr)_4_) and aluminium isopropoxide (Al(Pr)_3_), also represents an effective way to produce long-lasting and transparent nanoemulsions due to the surface-active properties of the catalysts, which help in the emulsion formation and stabilization [92]. In the case of biodiesel production, it should be noted that the production of stable emulsions, especially nanoemulsions, is strongly affected by the amount of alcohol and catalyst used in the biodiesel production system [142]. The addition of catalysts and alcohol beyond optimum values leads to undesirable effects, such as gel formation, and difficulties in phase separation. On the other hand, a relatively small amount of alcohol will affect the quality of micro-emulsion due to high viscosity [[148], [149], [150]]. In this way, supercritical fluids could also be applied along with ultrasonication and emulsification to increase the miscibility of both oil and aqueous phases [81].

Emulsification via cavitation-based technology has been also used for the conversion of heavy oils and vacuum residues. In particular, the addition of surfactants, such as nonylphenol with ethylene oxide (NO95: non-ionic), aerosol OT (AOT: anionic), benzalkonium chloride (BKC: cationic), and Tween 80 (non-ionic), assisted in the interaction of two immiscible phases [[119], [123]]. Upgrading petroleum residue mixture that consisted of toluene (to increase the flow of liquid), heated water (to increase the cavitation intensity), and aforementioned surfactants yielded an asphaltene reduction between 36 and 58 % depending on the type of processed vacuum residue [[81], [119]]. Another option that works as emulsion stabilizer is the addition of crude oil fractions, which in fact contain naturally occurring surface-active agents (e.g., naphthenic acids) [151].

It is worth mentioning that emulsions produced via cavitation methods are usually more stable compared with the ones created with conventional methods; more interestingly, it is able to produce droplets of smaller sizes, and efficient amounts of surfactants are required to stabilize the emulsion [21]. To separate the organic and aqueous phase after the process, microwave irradiation in a frequency range between 900 and 2500 MHz and centrifugation can be used as separation methods for the organic and aqueous phase, and in the case of nanoemulsions, which are highly stable systems, it may require to introduce de-emulsifying agents [[81], [147]]. As a preliminary concluding remark from this section, most of the studies miss to calculate or report the Reynolds number values (Re). On this basis, it would be possible to estimate the turbulence of the flow. Strategically, with the increase of the inlet pressure, the Reynolds and Weber number increases, and consequently, the cavitation number decrease, and small size droplets can be thus obtained [152].

## 6 Economic assessment for cavitation-based technologies

This section describes the cost of treatment, mainly in terms of energy efficiency for different reported cavitation processes in three groups of application of organic phase processes including desulfurization, heavy oil upgrade, and biodiesel production. HC is more energy-efficient compared with sonocavitation, being simpler in operation and less sensitive to geometric details of the reactor. Hence, it is needed to scale-up to meet industrial-scale operations and present better opportunities than ultrasonic reactors [[147], [153]].

The calculations are based on considering treatment time, the power of the system, and an average cost of electricity globally, which is 0.13 US Dollars (USD) per 1 kWh (3600 kJ) [154]. Among the cost of treatment, electrical energy per order E_EO_ (kWh/m^3^) was estimated as a uniform way of comparison. Electrical energy per order is described as the amount of energy needed to obtain a concentration change of the target compound by one order of magnitude (90 % change) in 1 m^3^ of processed fluid [155]. E_EO_ was calculated for the studies that reported a (%) yield of the reaction above 90 %. The EEO is calculated using any of the equations given below [156]:

(32) $$E_{\mathrm{EO}}=\frac{P_{\mathrm{el}}\;\times \;t\;\times 1000}{V\;\times \;60\;\times \;\log(\frac{\mathrm{Ci}}{\mathrm{Cf}})}$$

or

(33) $$E_{\mathrm{EO}}=\frac{38.4\times P_{\mathrm{el}}}{V\times k}$$

where P_el_ is power in (kW), t (min), V (L), C_i_ – initial concentration, and C_f_ – final concentration. Since Log (C_i_/C_f_) = k × t, equation 32 is transformed to equation (33).

The evaluation of the cost of treatment and process effectiveness is divided into two major groups including HC and acoustic cavitation (AC), in which both include the estimation of different organic phase processes (e.g., desulfurization, heavy oil upgrade, and biodiesel production).

**A. Organic phase processes based on hydrodynamic cavitation:**

Biodiesel production using HC represents the cheapest group of processes. The maximum treatment time needed during these processes was around 45 min. However, in some processes, even 5 min of processing was revealed to provide satisfactory results. Different types of orifices from single hole orifices to multi-hole orifices have been implemented in the systems of HC. The most cost-effective process was found to be the one using a 100-hole orifice (0.3 mm diameter), where 2.5 L waste cooking oil yielded 95 % biodiesel in 5 min treatment time, and an overall cost of the process was calculated as 4.8 $/m^3^ [42]. Using a single hole orifice, it increased the treatment time up to 45 min, which consequently raised the cost of treatment by approximately 10-fold [[154], [157]]. Concerning the desulfurization processes, the vortex diode was estimated to be the best cavitating device, yielding 100 % desulfurization (in 120 min treatment time) and a cost of the process of 36 $/m^3^ with an electrical energy per order (E_EO_) of 275 kWh/m^3^ [29]. The most expensive process for desulfurization was based on the usage of a single hole orifice (4 mm diameter), in which a 95 % desulfurization in 29 min resulted in a cost of treatment and E_EO_ of 52 $/m^3^ and 400 kWh/m^3^, respectively [59]. Even though the treatment time was relatively low, the reason for reporting the high cost of treatment is related to the electric power consumption of the system, where the electrical power consumption in the second study (ca. 4 000 W) was almost twice higher than the one reported in the first study (ca. 2200 W).

Concerning heavy oil upgrading aided by HC, both vortex diode and orifice were reported to be used as cavitation devices. Apparently, Vortex diode was found to be more effective for the treatment of 6 – 8 L heavy oil in the presence of gasoline as hydrogen donor, and 20 % viscosity reduction was achieved in 10 min treatment time. The total cost of treatment was calculated to be 8.9 $/m^3^ [30]. The use of orifice in the treatment of 50 g vacuum residue with kerosene, reached only 17.9 % viscosity reduction in 15 min treatment with a cost of approximately 490.6 $/m^3^ [78]. Therefore, the vortex diode is more effective in reducing the viscosity of heavy oil and vacuum residue.

**B. Organic phase processes through acoustic cavitation:**

From the economic point of view, acoustic cavitation is likely to be more expensive compared to HC. In the case of biodiesel production, the least expensive process corresponds to the use of a triple transducer system (20 kHz; 120 W) with the effectiveness of biodiesel as high as 95 %. Interestingly, 90 min processing had an overall cost of 6.7 $/m^3^ [88]. In such a process, increasing the ultrasonic power to 750 W (ultrasonic horn-based system) resulted in a cost of treatment of 2925 $/m^3^ and the treatment time was prolonged until 180 min [[137], [144]].

Concerning desulfurization processes, the lowest treatment cost was achieved for the desulfurization of 568.75 ppm diesel using an ultrasonic system (28 kHz; 200 W), where 93.3 % desulfurization was observed in 15 min treatment time with a cost of treatment and E_EO_ of 10.8 $/m^3^ and 83.3 kWh/m^3^, respectively [[88], [101]]. Lower ultrasonic power led to the longest treatment time, and as a consequence, less effective processes were obtained in terms of (%) desulfurization yield and cost of treatment. The treatment of 28 mL model fuel (containing 100 DBT in toluene) resulted in 32.56 % desulfurization in 90 min treatment time, while the treatment cost was found to be 433.3 $/m^3^ [[87], [100]]. The most effective device for the desulfurization of fuels by acoustic cavitation in terms of (%) yield, time (min), and cost of treatment, is an ultrasonic bath with a frequency of 28 kHz and power of 200 W, the least effective process was the use of a low ultrasonic power bath with the parameters of 35 kHz and 70 W, which concurrently required the longest time and higher treatment cost [100].

The cost of treatment of heavy oil upgrading was strongly dependent on the ultrasonic power. In this application, an ultrasonic probe with parameters of 20 kHz and 200 W was the cheapest method for the treatment of heavy oil, with a cost of 151.7 $/m^3^ [[31], [155]]. The process requiring 120 min sonication increased the yield of light oil, and the degree of cleavage in the thermal cracking of hydrocarbons. It is important to note that the most expensive process was obtained for an acoustic cavitation system, presenting an ultrasonic horn and transducers with a frequency of 20 kHz and ultrasonic power of 2000 W [[111], [122]]. An increase in gasoline and diesel yield, and a decrease in the viscosity of heavy oil, were obtained in 120 min. The cost of treatment was estimated to be approximately 2080 $/m^3^. Comparing effectiveness and prices for desulfurization and heavy oil upgrading, it can be concluded that in the first systems providing more unified sonocavitation in the fluid are desired, as reactions of desulfurization demand relatively lower energy input, while for heavy oil upgrading, effective cracking of hydrocarbons takes place in the ultrasonic probe, giving a relevant input of the energy instead probe immersion. For more detailed information, **Table 5S (**Supplementary material**)** reveals the main insights for all reviewed papers. Overall, it can be generalized that among the various cavitation-based organic phase treatment, the heavy oil upgrading was found to be more expensive as the constituents are very complex. The average time for biodiesel production using the cavitation process, utilizing an HC reactor presenting a 100-hole orifice (0.3 mm diameter), was 5 min and the estimated cost was 4.8 $/m^3^, which was cheaper than that of AC processes. Similarly, the cost of desulfurization processes using HC (with vortex diode) was estimated to be 36 $/m^3^, which is 10 times less than that of the AC process.

## 7 Current research gap and suggestion for new scientists in the field

Despite the current research starting to show advances, their hypothesis and planned studies are lacking in relevant insights due to omitting the evaluation, description and analysis of many important parameters, especially the ones having a significant impact on cavitation phenomena. For this reason, some crucial aspects of research on cavitation-based technologies are highlighted below hopping that future researchers will somehow study them:

A complete description of relevant details related to the geometry of the cavitation device is a must. A detailed description of the cavitation system is needed to be provided in terms of the materials and methods section of each research article. This will allow reproducing the studies by other researchers, make a fair comparison, as well as to calculate any needed parameters, such as linear velocity, etc. Secondly, such data will be useful as input for modelling the cavitation phenomenon. The scheme must include all dimensions and angles of the cavitation zone (such as vortex diode, Venturi slits), wherein the vortex diode, along with the angle of fluid injection to the cavitation zone, play a crucial role in the performance of the system in the case of HC process. In the case of the AC process, the material and shape of sonotrode should also be provided. There should be studies where Venturi can be implemented as a cavitation device since there is a lack of studies including the role of Venturi in experiments with the organic phase.

Most of the studies are focused on the characteristics of the main treated/processed organic liquid. However, in many studies, the second liquid phase is introduced as an aqueous stream of oxidants, additives, catalysts, among other reagents. It is evident that mass transfer between two non-miscible phases plays an essential role in the process kinetics. In many studies, the description of how an aqueous stream of reagent was introduced (and mixed) is not provided at all. In multiple studies, the processed liquid is pumped to a chamber/cavitation zone from a tank containing an organic phase and added (non-miscible) liquid reagent. Therefore, the generated phase separation makes the process very puzzling – which phase is pumped into the cavitation zone? How representative is the sample collected during the process? Post-process aqueous stream should be analyzed in terms of formed by-products, together with the main extracted components from treated feedstock, as well as secondary compounds produced inside reactions or via oxidation. Oxidized molecules often tend to dissolve in water. In the case of treatment processes such as desulfurization, it should be declared if removal was obtained by oxidation or simple extraction. The hazardous character of the aqueous stream should be evaluated, and proper treatment addressed. Processes that treat the organic phase and produce large quantities of aqueous strongly polluted effluents are not really “green” (or eco-friendly).

Studies on real feedstocks rather than on model two–three component model mixtures can provide more reliable results. However, to fully utilize this feature, the authors must provide as detailed as possible comprehensive characteristics of the feedstocks. In the case of cavitation-based processes, physiochemical characteristics (such as density, surface tension, and vapor pressure) are a must. However, chemical composition before/after the treatment makes data analysis more useful for persons investigating in the same field.

Oxidative processes in the organic phase, often tend to form compounds having higher hydrophilicity than primary compounds. If the goal is to remove some group of compounds, such as sulfur compounds. Future works should include an additional stage dedicated to extractive or sorptive removal of the by-products [33]. Such an attempt can easily assure the high final effectiveness of overall process. The cavitation process in organic phase treatment, especially the desulfurization can be compared with other oxidation processes, such as UV-H_2_O_2_ photolysis, Fenton process, photocatalysis, electrochemical AOPs, sulfate radical based AOPs, among others. In the case of photolytic, photocatalytic and electrochemical AOPs, their comparison could be made directly, i.e. these groups of processes are also feasible to be studied in an organic phase (non-aqueous) environment. Other AOP approaches can be used in bi-phasic systems, where an aqueous solution of oxidant is dispersed in an organic phase with the assistance of additional factors like UV light or catalysts. Finally, suggested in [33] approach – possible to be performed through cavitation phenomenon, could be separated into two stages where the first extraction of sulfur compounds would be performed, followed by their degradation in aqueous phase through AOPs.

The costs of treatment should be calculated together with its explained methodology, as it is considered one of the main evaluation aspects for implementing a method on a laboratory or industrial scale, and it would be found helpful for prospective studies. More reliable data, especially concerning energy costs, can be obtained for pilot-scale studies. On the contrary, large laboratory-scale units should be at least used instead of micro-scale reactors. In addition, cavitation is commonly known as a phenomenon causing severe damage to the pipelines. Most of the research takes a few months, therefore, it is advised to inspect the cavitation chamber after the treatment and document in the study if any sign of damage can be observed. This aspect is particularly relevant for planned scale-up of the process and continuous operation on a real industrial scale and in the estimation of cost.

## 8 Conclusion and future perspectives

According to this review, cavitation technologies are considered appropriate processes that fit into the idea of cleaner production, dealing with organic phase reactions in oil production such as desulfurization, heavy oil upgrading, reducing the viscosity of different oils, biofuel production, and emulsification. In comparison with AC-based processes, HC-based processes proved to be the most effective processes in terms of (%) yield, time (min), and also from the economical point of view for organic phase treatment.

The most useful cavitation unit in the above-mentioned applications was claimed to be the vortex diode, followed by a single hole orifice. It proved to give ≥ 95 % desulfurization yield and decreased the viscosity of heavy oils by 20 % in a short treatment time. As it corresponds to biodiesel production multiple holes orifices (specifically 100-hole orifice) was proved to give above 95 % biodiesel yield within 5 min process, and the cost of treatment consisted being the lowest one (4.8 $/m^3^) compared to all other organic phase applications based on HC and AC. Experimentally, higher efficiency was observed when the vortex diode was used compared with the orifice for the processes of heavy oil upgrading and desulfurization, meanwhile, the orifice predominates the efficiency in a biodiesel process application. The utilization of HC aided by hydrogen donors under optimized experimental conditions could lead to the conversion of heavy and high-molecular feedstock to lighter products.

The oxidants, such as H_2_O_2_, CH_3_COOH, HCOOH, Fenton reagent, nanomaterials, and their simultaneous use coupled with cavitation process, enhanced the desulfurization through the generation of peroxyl radical ^•^OOH, formil radical CHO^•^, acyl radical CH_3_CO^•^, and hydroxyl radical ^•^OH. Specifically, formil and acyl radicals are specific when formic acid and acetic acid were introduced as oxidizing agents.

HC installed in setup that ensures multicycle cavitation processes offer higher performance in upgrading heavy oil due to the maximum number of cavitation bubbles that get generated. To some extent, cavitational processing provides changes to the percentage of gasoline, naphtha, kerosene, and middle distillate fractions for different types of crude oil.

Regarding biodiesel production using cavitation processes, AC processes are the most used technique with an efficiency of approximately 95 %. However, concerning HC with only a few published studies, it was reported that the efficiency of HC reaches 99 % in biofuel production. The most used catalysts in transesterification processes were commonly KOH and NaOH. The combination of techniques, such as HC-microwave and HC-AC, would be an important approach for enhanced biofuel production.

## Declaration of Competing Interest

The authors declare that they have no known competing financial interests or personal relationships that could have appeared to influence the work reported in this paper.
