# Supplementary material for: Cavitation based cleaner technologies for biodiesel production and processing of hydrocarbon streams: A perspective on key fundamentals, missing process data and economic feasibility – A review
Source: Ultrason Sonochem. 2022 Jun 24;88:106081. doi: 10.1016/j.ultsonch.2022.106081 (PMC9253490; doi:10.1016/j.ultsonch.2022.106081)
Supplement: Supplementary data 1 [file mmc1.pdf]

<u>Supporting information</u>

Cavitation based cleaner technologies for biodiesel production and processing of hydrocarbon streams: A perspective on key fundamentals, missing process data and economic feasibility – a review

Elvana Cako[1], Zhaohui Wang [2,3,4], Roberto Castro-Muñoz[5,6], Manoj P. Rayaroth[1,7], Grzegorz Boczkaj[5,8]*

[1*]Department of Process Engineering and Chemical Technology, Faculty of Chemistry, Gdańsk University of Technology, Poland.

[2]Shanghai Key Lab for Urban Ecological Processes and Eco-Restoration, School of Ecological and Environmental Sciences, East China Normal University, Shanghai 200241, China.

[3]Institute of Eco-Chongming (IEC), No.20 Cuiniao Road, Chen Jiazhen, Shanghai 202162, China.

[4]Technology Innovation Center for Land Spatial Eco-restoration in Metropolitan Area, Ministry of Natural Resources, 3663 N. Zhongshan Road, Shanghai 200062, China.

[5] Department of Sanitary Engineering, Faculty of Civil and Environmental Engineering, Gdańsk University of Technology, Poland.

[6]Tecnologico de Monterrey, Campus Toluca. Av. Eduardo Monroy, Cárdenas 2000 San Antonio Buenavista, 50110 Toluca de Lerdo, Mexico

[7] GREMI, UMR 7344, Université d'Orléans, CNRS, 45067 Orléans, France

[8]EkoTech Center, Gdansk University of Technology, G. Narutowicza St. 11/12, 80-233 Gdansk, Poland

**Corresponding author: Prof. Grzegorz Boczkaj, PhD. Sc. Eng. Gdansk University of Technology, Faculty of Civil and Environmental Engineering, Department of Sanitary Engineering, 80 – 233 Gdansk, G. Narutowicza St. 11/12, Poland. Fax: (+48 58) 347-26-94; Tel: (+48) 697970303; E-mail: grzegorz.boczkaj@pg.edu.pl*

Table Captions:

Table S1: Hydrodynamic cavitation based organic phase processing.

Table S2: Effectiveness of acoustic cavitation in the desulfurization of diesel, gasoline, and different model fuels.

Table S3: Heavy oil upgrading utilizing acoustic cavitation.

Table S4: Acoustic cavitation based biodiesel production.

Table S5: Economic aspects of cavitation based organic phase processing.

Table S1: Hydrodynamic cavitation based organic phase processing.

Nr	**Reference**	**Equipment**	**Sample**	**Oxidants/ Catalysts**	**Extraction solvent**	**Parameters**	**Cavitation number**	**(%) Yield**
1	Suryawanshi et al., [1]	Hydrodynamic cavitation/vortex diode (Desulfurization)	300 ppm model sulfur of Thiophene in octane; n-octanol; toluene	-	-	Flow rate L/h: 330 and 630 Pressure[bar]: 0.5 and 2 V (L): 12-20 Time (min):120	Assumed cavitation number a) 0.5 bar and 330L/h: octane (C_v=2.53) n-octanol (C_v=2.16) toluene (C_v=2.02) b) 2 bar and 630L/h: octane (C_v=1.19) n-octanol (C_v=1.01) toluene (C_v=0.96)	100% desulfurization
2	Baradaran and Sadeghi [2]	Hydrodynamic cavitation/single	Non-hydro treated diesel	Formic acid/H_2O_2	a) Washing stage with	Time(min):29 V(L): 5	Cavitation number (-)	95% desulfurization

		hole orifice (Desulfurization)	1 750 ppm	n_a/n_o (1-5) Optimal n_a/n_o=3.2	water for 15 min b) Mixing with acetonitrile for 15 min at 500 rpm	Temp(°C) =50±2 Pressure[bar]: 4.2		
3	Suryawanshi et al., [3]	Hydrodynamic cavitation/orifice (Desulfurization)	100-300 ppm Thiophene in octane; n-octanol; toluene	-	-	Time(min):120 V(L):12-20 Pressure[bar]: 0.5 bar for vortex diode and 2 bar for orifice Flow rate L/h: 390, 560,785	Assumed cavitation number: Toluene (Cv=1.42 flow 390 L/h; Cv=0.69 flow 560 L/h; Cv=0.35 flow 785) n-Octanol (Cv=1.53 flow 390 L/h; Cv=0.74 flow 560 L/h; Cv=0.37 flow 785 L/h) Octane (C_v=1.79	95% desulfurization

							flow 390 L/h; C_v=0.86 flow	
4	Askarian et al.,[4]	Hydrodynamic cavitation/ Vortex diode (Heavy oil upgrade)	Heavy oil fuel; Silicone oil for an experiment to investigate parameters	Gasoline as hydrogen donor	-	Temp(°C): 80 Flow rate L/min: 16 Time(min): 15 V[L]: 6-8	Cv 0.08	19 % Viscosity reduction
5	Ansari et al., [5]	Hydrodynamic cavitation/orifice (Heavy oil upgrade)	Crude oil Vacuum residue: Kerosene (68:32)			Temp(°C): 30;50 Time(min):15 Flow rate L/h:150-750 (2.5 – 12.5 L/min) (crude oil) and 830 L/h (13.8 L/min) (kerosene)	Optimal cavitation number C_v=0.15	Crude oil: 836.50 kg/m^3 before cavitation- 831 kg/m^3 after cavitation Viscosity 0.00666 Pa S before cavitation

						Pressure: 0.5-0.8MPa (crude oil) and 0.5-0.7 MPa(kerosene)		0.00663 Pa S after cavitation VR+ kerosene: 940.94 kg/m^3 before cavitation- 937.42 kg/m^3 after cavitation Viscosity 0.173 Pa S before cavitation and 0.142 Pa S after cavitation 17.9 % viscosity reduction
6	Wan et al.,[6]	Cavitating jet/cavitation nozzle	Heavy oil	THN (tetrahydro naphtalene)	-	Flow rate L/min: 75 Pressure drop:	Assumed cavitation number 2.11	Viscosity reduction rate increased to 11.2

		(Heavy oil upgrade)		as hydrogen donor		40 MPa		and 15.7%
7	Hilares et al., [7]	Hydrodynamic cavitation/orifice (Biofuel production)	Sugarcane bagasse	(0.29 M) $NaOH/H_2O_2$(0.78 % v/v)	-	Flowrate m^3/h: 5 Velocity through orifice m/s: 88 Pressure[bar]:3 Temp(°C): 60 V[L]:3 Time(min):2-10	Optimal cavitation number: 0.017	>95% hydrolysis yield
8	Chuah et al.,[8]	Hydrodynamic cavitation/orifice (Biofuel production)	Waste cooking oil (Palm olein)	1 wt% KOH: Methanol (0.5-1.25wt%)	-	Oil-methanol molar ratio (1:4-1:7) Temp(°C):50-65 Pressure[bar]:2 V[L]: 6.2 Time(min):15	Assumed cavitation number: 0.065	98.1% methyl ester conversion
9	Hilares et	Hydrodynamic	Sugarcane	Na_2CO_3	-	Time(min):20	Reported	95% Yield in

	al.,[9]	cavitation/orifice (Biofuel production)	bagasse	0.5, 1.0, 1.5 mol/L; $Ca(OH)_2$ 0.5 mol/L; KOH 0.1, 0.3 and 0.5 mol/L; NaOH 0.3 mol/L		V(L):3 Upstream pressure[bar]:3 Downstream pressure[bar]:0.3	cavitation number 0.07	enzymatic hydrolysis of pretreated SCB
10	Yadav et al.,[10]	Hydrodynamic cavitation/orifice (Biofuel production)	Yellow Oleander (Thevetia Peruviana) Oil	KOH (1 wt%) dissolved in Methanol 6:1 Oil to methanol molar ratio	-	Temp(°C):40-55 V(L): 5 Time(min):30-45	-	97.5% transesterificatio n

11	Bargole et al.,[11]	Hydrodynamic cavitation/ 3 different Orifices OP1, OP2, OP3 /circular Venturi	Waste cooking oil	KOH dissolved in methanol	-	Temp(°C): 35±2 V(L): 2.5 Time(min):18, 15, 15,5 Pressure [Bar]: 3, 5, 7, 10, 15	Optimal Cavitation number: CV (1.07, 0.52, 0.30, 0.19, 0.08) OP1(1.56, 0.90, 0.49, 0.22, 0.12, 0.12) OP2(1.17, 0.63, 0.46, 0.28, 0.21) OP3(0.98, 0.55, 0.34, 0.23, 0.11)	99% trasnesterification yield for OP3 and 7 bar (5 min): 76% transesterification yield for OP2 and 7 bar (5 min) 87% transesterification yield for OP1 and 7 bar (5 min) 75 % transesterification yield for circular Venturi (5 min)

12	Kelkar et al.,[12]	Hydrodynamic cavitation	Fatty acids odour cut (C_8-C_{10})	Methanol: H_2SO_4	-	Temp(°C): 28<br>Time(min): 180<br>V(L):10	-	>90% biodiesel yield
13	Mohod et al.,[13]	Hydrodynamic cavitation/ high speed homogenizer	Waste cooking oil, fresh cooking oil	KOH 3 wt%; Methanol: oil ratio (12:1)	-	Temp(°C): 50<br>Time(min): 120<br>V(L): -	-	97% biodiesel yield for waste cooking oil as starting material and 92.3% for fresh cooking oil
14	Maddikeri et al.,[14]	Hydrodynamic cavitation/ Orifice plate, circular and slit venturi as cavitation device	Waste cooking oil	KOH 1.0 wt%<br>Oil to methyl acetate ratio 1:12	-	Temp(°C): 50<br>Time(min): 60<br>V(L): 15 L (holding tank)<br>Pressure [Bar]: 3	Assumed cavitation number: C_v=0.33	90% biodiesel yield (Slit Venturi)<br>82% biodiesel yield (Circular Venturi)<br>64% biodiesel yield (single hole orifice)

Table S2: Effectiveness of acoustic cavitation in the desulfurization of diesel, gasoline, and different model fuels.

Nr	Reference	Sample	Freq. (kHz)	Power (W)	Oxidant/Catalysts	Time (min)	T (°C)	%S removal	V(mL)
[1]	Dai et al.,[15]	Diesel fuel (568.75 ppm)	28	200	Fenton salt; H_2O_2 (0.5 mol/mol)	15	40	98.3	100-600
[2]	Suryawanshi et al., [16]	TH; BT; DBT in n-octane (100 ppm)	33	250	Shirasagi-TAC; CFP-450 with combination of different metal catalysts (Zn;Co; Ni; Cu)	15	28	100	10
[3]	More and Gogate [17]	Thiophene in n-hexane (300 ppm)	20	100	H_2O_2; TiO_2; UV	60	60	99	500
[4]	Ramos et al.,[18]	DBT in hexadecane (300 ppm)	42	185	FeOx/SBA-15 FeOx/Zr-SBA-15	30	60	100	100
[5]	Shayegan et al.,[19]	Gas oil (9500 mg/kg)	24	400	$FeSO_4$; H_2O_2; tetraoctyl ammonium bromide	17	62 ± 2	90	30

[6]	Duarte et al.,[20]	4, 6 DMDBT in toluene (180 ppm)/ Diesel (136 ± 5 ppm)	20	750	H_2O_2; Acetic Acid	9	90	98 (model fuel) 75 (diesel oil)	25
[7]	Zhao et al.,[21]	Model diesel	20	150	CdO; H_2O_2; Acetic Acid	180	50	99.47	30
[8]	Mei et al.,[22]	DBT in toluene (400 ppm); Diesel (A: 7 744, B: 3 011 and C: 1867 ppm)	20	600	H_2O_2; phosphotungstic acid; tetraoctyl ammonium bromide	10	75	99.4 (Diesel C)	50
[9]	Desai et al.,[23]	DBT in n-octane (500 ppm)	20	250	H_2O_2; Acetic Acid PTC (phase transfer catalyst); triphenyl phosphonium bromide	30	50	75.23	25
[10]	Lin et al., [24]	Crude oil (2133 ppm)	70	100	H_2O_2; Phosphotungstic acid; Sb_2O_5; Active Ni	10	65	65.28	-
[11]	Nunes et al.,[25]	Petroleum product feedstock containing s-compounds and	20	750	Acetic Acid; H_2O_2	5	-	95	25

		nitrogen (226 and 376 ppm)							
[12]	Afzalinia et al.,[26]	BT; DBT; 4,6 DMDBT in n-octane (400 ppm)	37	50-150	Keggin type phosphotungstic acid ($H_3PW_{12}O_{40}$, PTA) encapsulated into an amino functionalized MOF (TMU-17-NH2)	15	20-25	98	10
[13]	Carnaroglio et al.,[27]	DBT; 4,6 DMDBT in toluene (1.2 mg/mL); Hydrotreated diesel feedstock (S: 226 ± 2.17 ppm and N: 158 ± 2.81 ppm)	19.9; 21.1	80	Oxone; sodium persulfate; potassium superoxide	30	80	>95	25
[14]	Wang et al.,[28]	Th; BT; DBT;in n-octane; fuel	-	300	Nickel-heteropolyacids supported on the silica gel	40	40	100	100
[15]	Wan and Yen [29]	Marine gas oil	20	600	Tetraoctyl ammonium fluoride;	10	71	98.8	42

		(1 710 ppm)			H_2O_2; phosphotungstic acid				
[16]	Akbari et al.,[30]	BT and DBT in n-hexane (1 000 ppm)	20	300	MoO_3/Al_2O_3; H_2O_2	30	45	98	50
[17]	Margeta et al.,[31]	DBT in n-heptane; n-dodecane; n-hexadecane (3 976.86 ppm)	20	70	H_2O_2; Acetic acid	30	70	98.35	70
[18]	Bhasarkar et al.,[32]	DBT in toluene 100 ppm	35	70	Acetic acid; formic acid; Tetraoctylammonium bromide	90	25	47.05	28
[19]	Gonzalez et al.,[33]	Middle distillate fuels (Jet fuel)	20	200	Formic acid; H_2O_2; activated carbon	120	65	98	36
[20]	Tang et al., [34]	Bunker-C oil	20	240	Tert-butyl hydroperoxide/ Molibdenum oxide system	90	80	35	25
[21]	Choi et al., [35]	BT; DBT in toluene (500 ppm)	20	200	Polyoxometalate catalysts $Na_3PW_{12}O_{40}$; $H_3PW_{12}O_{40}$; $H_3PM_{12}O_{40}$; $H_4SiW_{12}O_{40}$	30	30-70	94.8	160
[22]	Bhasarkar et al., [36]	DBT in toluene 100 ppm	35	70	Acetic acid; H_2O_2; Fenton salt	90	25	32.56	38

[23]	Zhou et al., [37]	Gasoline	10-21	400	Formic acid; H_2O_2	7	50	80.87	50
[24]	Bolla et al., [38]	TH; 3-methyl thiophene; BT in n-hexane (100, 300 and 500 ppm)	35	35	Acetic Acid; H_2O_2; Fenton salt	90	25	77.5; 77.6; 77.9	25
[25]	Abdi et al., [39]	DBT in n-dodecane (100,200,300,500, 800, 1000 ppm)	20	120	GO-COOH (highly acidic and modified catalyst using chloroacetic acid)	300	25; 40	95	30
[26]	Ammar et al., [40]	Model fuel with 200 mg/L pyrrole and indole	19.85 - 20.05	400	graphene oxide supported with phosphomolybdic acid (PMo-Fe3O4/rGO)	240	30		50

Table S3: Heavy oil upgrading utilizing acoustic cavitation.

Nr	Reference	Sample	Freq (kHz)	Power (W)	Equipment	Catalysts/Oxidants	T(°C)	T(min)	V(mL)	Parameters after cavitation
1	Song et al.,[41]	Residual oil	20	200	High pressure stirring autoclave and ultrasonic probe type device	-	400-440	120	240	%wt Fractions yield: Gasoline 26.54%; Diesel 55.27%; VGO 6.95%; Residual oil yield 2.74%
2	Montes et al., [42]	Heavy crude oil	37	400	Elmasonic E60H sonicator	$NiO-SiO_2$ nanoparticles	25	120	-	50-60% viscosity reduction
3	Mirzoeva et al.,[43]	Coker naphta IBP-85°C	26	44	Hielsher UP 200 ST ultrasonicator	-	80	50	-	The yield of liquid products 3.6%; olefins 12.1%; alkyl ethyl esters 5.6 %;

										the octane rating of the etherification product increased by 6.0 MON points or 9 RON points; catalyst coking decreased by 50–55%
4	Fan et al.,[44]	Tar-sand bitumen	20	2 000	An ultrasonic reactor in the presence of ultrasonic probe, ultrasonic transducer and ultrasonic horn	-	400-440	120	250	%wt Fractions yield: Gasoline 28.9%; Diesel 61.1%, VGO 9.5%, Residual oil 0.5%
5	Gopinath et al.,[45]	Heavy gas oil	20	1 500	Ultrasonic probe	-	400	20	200	11% nitrogen conversion and 7%

										sulfur conversion
6	Kaushik et al.,[46]	Vacuum residual feedstock (Asphaltene: 13.5 wt%) (S:5.77 wt%)	24	200	Ultrasonic probe	Non-ionic surfactant (Tween 80)	30	90	50	Vacuum residue decreased from 13.5 (initial asphaltene present in residue) to 7% (wt.%). 48% asphaltene reduction.
7	Khodaei et al.,[47]	Non-hydrotreated kerosene (2 490 ppm)	20	250	Titanium ultrasonic probe	HCOOH; H_2O_2 no/ns = 15.02, n acid/n S = 107.8	50	10.5	50	95.46% sulfur removal from kerosene
8	Cui et al.,[48]	Crude oil	20	-	Ultrasonic cell pulverizer	Nickel oleate catalyst	-	20	25 g	Increased amount of lighter components and reduced viscosity
9	Shi et al.,[49]	Heavy oil	28	1800	Autoclave ultrasonic static mixer	-	360	30		57.34% viscosity reduction

	reactor<br><br>(Transducer)	

Table 4: Acoustic cavitation based biodiesel production.

Nr	Reference	Equipment	Sample	Catalysts/ oxidants	Temp (°C)	V(L)	Time (min)	%Yield
1	Chemat et al.,[50]	Sonoprobe (20 kHz;150 W)	Sunflower oil	-	20	0.1 L	0.5-2	Peroxide value (PV) was increased from 5.38 meq. O_2/kg oil for untreated oil to 6.33 meq. O_2/kg oil for sunflower oil
2	Stavarache et al., [51]	Ultrasonic Cleaners (28-40 kHz; 1200 W)	Vegetable oil	KOH; NaOH dissolved in alcohol (Methanol) MeOH:oil=6:1	20	0.1 L	10-20	98-99% transesterification of fatty acids for 28 kHz
3	Kashyap et al., [52]	Ultrasonic horn (20 kHz and 120 W)	Karanja oil	KOH (5.25 wt%) Methanol:oil (10:1)	60	0.1 L	35	91.5% FAME yield
4	Kelarijani et al., [53]	Ultrasonic bath (37 kHz and 1 000 W)	Rapeseed oil	Nanomagnetic catalysts Li/Fe_3O_4 and Li/ZnO–Fe_3O_4	35	-	35	99.8% Biodiesel yield

5	Korkut et al., [54]	Ultrasonic horn (20 kHz and 200 W)	Canola oil	Methanol (methanol: oil ratio 7.48) CaO, calcined dolomite, and calcium diglyceroxide (CaDG)	60	0.25 L	150	99.4% biodiesel yield in case of CaO catalysts
6	Nikseresht et al., [55]	Ultrasonic bath (37 kHz and 50 W)	Oleic acid	Fe(III)-based MOF, namely MIL-53 (Fe) Phosphotungstic acid Ethanol Butanol	20-25	0.12 L	15	96% biodiesel yield in case of catalysts loading PTA@MIL-53 (Fe) and ethanol; 98% biodiesel yield in case of catalyst loading PTA@MIL-53 (Fe) and n-butanol.
7	Hingu et al., [56]	Ultrasonic horn (20 kHz and 200 W)	Waste cooking oil	KOH; Methanol	45	0.1 L	40	89.5% transesterification yield
8	Sarve et al., [57]	Ultrasonic horn (20 kHz; 1	Sesame oil	$Ba(OH)_{2;}$ Methanol	25-30	0.25 L	40.30	98.6% FAME yield

		200)						
9	Prakash Maran et al., [58]	Ultrasonic bath (20 kHz; 400 W)	Muskmelon oil	KOH; Methanol	30	0.3 L	30	97.56 ± 0.63% FAME conversion yield
10	Subhedar et al., [59]	Ultrasonic horn (20 kHz; 120 W)	Waste cooking oil	Thermomyces lanuginosus (Lipozyme TLIM) enzyme as a catalyst	50	0.1 L	30	96.1% biodiesel yield
11	Michelin et al., [60]	Ultrasonic horn (20 kHz; 132 W)	Macauba coconut oil	NaOH; ethanol Commercial immobilized lipase, Novozym 435, from Candida Antarctica	60	0.02L	90	90% conversion yield
12	Rocha et al., [61]	Ultrasonic bath (40 kHz; 160 W)	Spent coffee grounds oil	KOH; Methanol	60	0.5 L	45	0.5 g ethanol/ g glucose; productivity of 1.9 g/L*h
13	Maddikeri et al., [62]	Ultrasonic horn (22 kHz; 750 W)	Waste cooking oil	KOH; Methanol	40	0.1 L	180	90% biodiesel yield

14	Manickam et al., [63]	Ultrasonic transducers (multiple frequencies 28, 40, 70 kHz; 300 W)	Palm oil	KOH; Methanol where methanol: oil ratio 9:1	60	0.1L	120	93% biodiesel yield
15	Kelkar et al., [12]	Ultrasonic bath with three transducers (20 kHz; 120 W)	Fatty acids cut (C_8-C_{10})	Methanol; H_2SO_4 Methanol:fatty acids cut (5:1 or 10:1)	28 ± 2	3.5 l	90	>95% esterification yield

** In the case of reference paper 7, 9, 11 and 14 the amount of oil was converted to L. The densities for each oil are given below:

Hingu et al., ((100 g WCO wate cooking oil; density (925 kg/m^3)); Prakash Maran et al., ((300 g Muskmelon oil; density (892 kg/m^3 [64])); Michelin et al., ((20 g Macauba coconut oil; density 927 kg/m^3)); Manickam et al., ((100 g Palm oil; density 879 kg/m^3))

Table 5: Economic aspects of cavitation based organic phase processing.

Nr	Ref.	Study	Process	Time[min]	V[L]	Power [kW]	Energy consumption [kWh]	Energy consumption [kJ]	Cost of Energy [USD]	Cost of treatment [USD/m^3]	E_{EO} [kWh/m^3]	(%yield)
**1**	Dai et al., [15]	Acoustic cavitation (20 kHz; 200 W) Home-made ultrasonic bath	Desulfurization (Diesel fuel; 568.75 ppm)	15	0.6	0.2	0.05	180	0.0065	10.8	83.3	98.3
**2**	Suryawanshi et al., [16]	Acoustic cavitation (33 kHz;	Desulfurization (Th, BT, DBT	15	0.01	0.25	0.06	225	0.0081	812.5	6 250	100

		250 W) Ultrasonic bath	in n-octane; 100 ppm)									
**3**	More and Gogate [65]	Acoustic cavitation (20 kHz; 1050 W) Ultrasonic flow cell	Desulfurization (100-500 ppm thiophene in n-hexane)	80	0.5	1.05	1.4	5040	0.182	364	2 800	96
**4**	Duarte et al., [20]	Acoustic cavitation (20 kHz; 750 W) Ultrasonic probe	Desulfurization (4,6- DMDBT in toluene; 180 ppm)	9	0.025	0.75	0.18	675	0.0143	585	4 500	98
**5**	Mei et al.,[22]	Acoustic cavitation (20 kHz; 600 W)	Desulfurization (Diesel; 1867 ppm)	10	0.05	0.6	0.10	360	0.013	260	2 000	99.4

		Titanium ultrasonic probe										
**6**	Desai et al.,[23]	Acoustic cavitation (20 kHz; 250 W) Titanium ultrasonic probe	Desulfurization (DBT in n-octane; 500 ppm)	30	0.025	0.25	0.125	450	0.0162	650	-	75.23
**7**	Nunes et al., [25]	Acoustic cavitation (20 kHz; 750 W) Titanium ultrasonic probe	Desulfurization (Petroleum product feedstock with S:226 ppm and N: 376 ppm)	5	0.025	0.75	0.06	225	0.0081	325	2 500	95

**8**	Afzalinia et al., [26]	Acoustic cavitation (50 kHz; 150 W) Ultrasonic bath	(Desulfurization 500 ppm BT, 4,6 DMDBT, BT in n-octane)	15	0.010	0.15	0.05	180	0.0065	487.5	3 750	98
**9**	Carnarogli o et al., [27]	Acoustic cavitation (19.9 kHz, 21.1 kHz, 80 W) Ultrasonic horn	Desulfurization/ Denitrification (Hydrotreated diesel feedstock S: 226 ± 2.17 ppm; N: 158 ± 2.81 ppm)	30	0.025	0.08	0.04	144	0.0052	208	1 600	>95
**10**	Wan and Yen.,[29]	Acoustic cavitation (20 kHz; 600 W)	Desulfurization (Marine gas oil; 1710 ppm)	10	0.042	0.6	0.10	360	0.0130	309.5	2381.0	98.8

		Ultrasonic probe										
**11**	Akbari et al., [30]	Acoustic cavitation (20 kHz; 300 W) Ultrasonic probe	Desulfurization (BT; DBT in n-hexane; 1000 ppm)	30	0.01	0.30	0.9	3 240	0.117	1 170	9 000	98
**12**	Margeta et al., [31]	Acoustic cavitation (20 kHz; 70 W) Ultrasonic apparatus	Desulfurization (DBT in n-heptane; 3976 ppm)	30	0.07	0.07	0.175	630	0.0227	325	2 500	98.35
**13**	Bhasarkar et al., [66]	Acoustic cavitation (35 kHz; 70 W)	Desulfurization (DBT in toluene: 100	90	0.038	0.07	0.105	378	0.0136	487.5	-	47.05

		Ultrasonic bath	ppm)									
**14**	Gonzalez et al., [33]	Acoustic cavitation (20 kHz; 200 W) Ultrasonic apparatus	Desulfurization (JP-8 Jet fuel with S: 717 ppm)	120	0.036	0.2	0.4	1 440	0.052	1 444.4	11 111	98 for jet fuel; 94 for diesel; 88 for ultralow-sulfur diesel
**15**	Bhasarkar et al., [36]	Acoustic cavitation (35 kHz; 70 W) Ultrasonic bath	Desulfurization (DBT in toluene: 100 ppm)	90	0.038	0.07	0.105	378	0.0693	433.3	-	32.56
**16**	Choi et al.,[35]	Acoustic cavitation	Desulfurization	30	0.16	0.20	0.101	360	0.013	81.3	625	94.8

		(20 kHz; 200 W) (Titanium ultrasonic probe)	(500 ppm BT in toluene; 500 ppm DBT in toluene)									
**17**	Zhou et al., [37]	Acoustic cavitation (10-21 kHz; 700 W) Ultrasonic probe	Desulfurization (Gasoline 0.1207 wt%; Crude oil 2.862 wt% S-content)	10	0.1	0.7	0.117	420	0.0136	487.5	-	80.87
**18**	Bolla et al.,[38]	Acoustic cavitation (35 kHz; 35 W) Ultrasonic bath	Desulfurization (TH, 3-methylthiophene, BT in n-hexane; 100/300/500	90	0.025	0.035	0.052	189	0.0068	273.0	-	≈ 78

			ppm)									
**19**	More and Gogate [67]	Acoustic cavitation (20 kHz; 100 W; 2 UV lamps 8 kW each) Ultrasonic horn	Desulfurization (TH in n-hexane: 300 ppm)	60	0.5	0.116	0.116	417.6	0.0150	30.16	232.0	99
**20**	Song et al., [41]	Acoustic cavitation (20 kHz; 200 W) Ultrasonic probe	Heavy oil upgrade	120	0.24	0.2	0.4	1 440	0.0151	151.7	-	(Table 3)
**21**	Fan et al., [44]	Acoustic cavitation (20 kHz; 2	Heavy oil upgrade	120	0.25	2	4	14 400	0.5210	2 080	-	(Table 3)

		000 W) Ultrasonic probe; transducer; ultrasonic horn										
**22**	Gopinath et al., [45]	Acoustic cavitation (20 kHz; 1 500 W) Ultrasonic probe	Heavy oil upgrade (Model S-compound 1600 ppm and Model N-compound 6000 ppm)	20	0.20	1.5	0.5	1 800	0.0650	325	-	(Table 3)
**23**	Kaushik et al., [46]	Acoustic cavitation (24 kHz; 200 W) Ultrasonic	Heavy oil upgrade	90	0.05	0.2	0.3	1 080	0.0390	780	-	(Table 3)

		probe										
24	Khodaei et al., [47]	Acoustic cavitation (20 kHz; 380 W) Titanium ultrasonic probe	Heavy oil upgrade (2490 ppm S-content kerosene)	10.5	0.05	0.38	0.066	239.4	0.0086	823	1 330	95.46
25	Chemat et al., [50]	Acoustic cavitation (20 kHz, 150 W) Titanium ultrasonic probe	Biodiesel production (Sunflower oil)	30	0.1	0.15	0.075	270	0.0097	98	-	(Table 4)
26	Stavarache et al., [51]	Acoustic cavitation (28-40 kHz;	Biodiesel production (Vegetable	20	0.1	1.2	0.401	1 440	0.0521	520	-	99.8

		1200 W) Ultrasonic horn	oil)									
**27**	Kashyap et al., [52]	Acoustic cavitation (20 kHz; 120 W) Ultrasonic horn	Biodiesel production (Karanja oil)	35	0.1	0.12	0.070	252	0.0091	91	-	91.5
**28**	Korkut and Bayramoglu [54]	Acoustic cavitation (20 kHz; 200 W) Ultrasonic horn	Biodiesel production (Canola oil)	150	0.25	0.20	0.501	1800	0.065	260	-	99.4
**29**	Nikseresht et al.,[55]	Acoustic cavitation (37 kHz; 50 W)	Biodiesel production (Oleic acid)	15	0.12	0.05	0.013	45	0.0016	13.5	-	96.98

		Ultrasonic bath										
**30**	Sarve et al.,[57]	Acoustic cavitation (20 kHz; 1 200 W) Ultrasonic bath	Biodiesel production (Sesame oil)	40	0.25	1.2	0.800	2880	0.104	416.0	-	98.6
**31**	Prakash Maran et al., [58]	Acoustic cavitation (20 kHz; 400 W) Ultrasonic horn	Biodiesel production (Muskmelon oil)	30	0.3	0.4	0.2	720	0.026	86.7	-	97.56
**32**	Subhedar and Gogate [59]	Acoustic cavitation (20 kHz; 120 W) Ultrasonic	Biodiesel production (Waste cooking oil)	180	0.1	0.12	0.36	1296	0.0468	468.0	-	96.1

		horn										
**33**	Michelin et al.,[60]	Acoustic cavitation (20 kHz; 132 W) Ultrasonic horn	Biodiesel production (Macauba coconut oil)	90	0.02	0.132	0.198	712.8	0.0257	1287	-	90
**34**	Rocha et al.,[61]	Acoustic cavitation (40 kHz; 160 W) Ultrasonic bath	Biodiesel production (Coffee ground seed oil)	45	0.5	0.16	0.12	432	0.0156	31.2	-	97
**35**	Maddikeri et al.,[68]	Acoustic cavitation (20 kHZ; 750 W) Ultrasonic	Biodiesel production (Waste cooking oil)	180	0.1	0.75	2.25	8100	0.2925	2925	-	90

		horn										
**36**	Kelkar et al.,[69]	Acoustic cavitation (20 kHz; 120 W) Ultrasonic bath with three transducers	Biodiesel production (Fatty acids cuts)	90	3.5	0.12	0.18	648	0.0234	6.7	-	95
**37**	Suryawanshi et al., [70]	Hydrodynamic cavitation (Power 2 200 W) Vortex diode	Desulfurization (500 ppm of Thiophene in n-octane; n-octanol; toluene)	120	20	2.2	5.5	19 800	0.7150	36	275	100
**38**	Suryawanshi et al.,	Hydrodynamic	Desulfurization	120	20	2.2	6.6	23 760	0.8580	42.9	330	95

	[3]	cavitation (Power 2 200 W) Vortex Diode; orifice	(100-300 ppm Thiophene in n-octane; n-octanol; toluene)									
**39**	Baradaran and Sadeghi [71]	Hydrodynamic cavitation (Power 4 000 W) Single hole orifice	Desulfurization (1750 ppm Diesel feedstock)	29	5	4.0	2.0	7200	0.26	52.0	400	95
**40**	Ansari et al., [72]	Hydrodynamic cavitation (Power 800 W) Orifice	Heavy oil upgrade	15	0.053	0.8	0.2	720	0.026	490.6	-	(Table 1) 17.9 % viscosity reducti

												on
**41**	Askarian et al.,	Hydrodynamic cavitation (Power 2 200 W) Vortex diode	Heavy oil	10	6-8	2.2	0.55	1980	0.0715	8.9	-	(Table 1) 20% viscosity reduction
**42**	Hilares et al., [73]	Hydrodynamic cavitation (Power 1 500 W) Orifice	Biodiesel production (Sugarcane bagasse)	10	3.0	1.5	0.25	900	0.0325	10.8	-	95
**43**	Chuah et al., [74]	Hydrodynamic cavitation (Power 4	Biodiesel production (Palm oil)	15	6.2	4.4	1.1	3 960	0.1431	23.1	-	98

		400W) Orifice										
**44**	Bargole et al., [75]	Hydrodynamic cavitation (Power 1 100 W) Three different orifice; circular Venturi	Biodiesel production (Waste cooking oil)	5	2.5	1.1	0.33	1 188	0.0429	4.8	-	99
**45**	Yadav et al.,[10]	Hydrodynamic cavitation (Power 2 200 W) Orifice	Biodiesel production (Yellow oleander oil)	45	5	2.2	1.65	5940	0.2145	42.9	-	97.5

**46**	Maddikeri et al.,[14]	Hydrodynamic cavitation (Power 1 500W) Single hole orifice; circular venturi; slit Venturi	Biodiesel production (waste cooking oil)	120	15	1.5	3	10800	0.39	26.0	-	90 (slit Venturi)

[1] N.B. Suryawanshi, V.M. Bhandari, L.G. Sorokhaibam, V. V. Ranade, A Non-catalytic Deep Desulphurization Process using Hydrodynamic Cavitation, Sci. Rep. 6 (2016) 1–8. doi:10.1038/srep33021.

[2] S. Baradaran, M.T. Sadeghi, Intensification of diesel oxidative desulfurization via hydrodynamic cavitation, Ultrason. Sonochem. 58 (2019) 104698. doi:10.1016/j.ultsonch.2019.104698.

[3] N.B. Suryawanshi, V.M. Bhandari, L.G. Sorokhaibam, V. V. Ranade, Developing techno-economically sustainable methodologies for deep desulfurization using hydrodynamic cavitation, Fuel. (2017). doi:10.1016/j.fuel.2017.08.106.

[4] M. Askarian, A. Vatani, M. Edalat, Heavy oil upgrading via hydrodynamic cavitation in the presence of an appropriate hydrogen donor, J. Pet. Sci. Eng. 151 (2017) 55–61. doi:10.1016/j.petrol.2017.01.037.

[5] K.B. Ansari, N.H. Loke, A.B. Pandit, V.G. Gaikar, R. Sivakumar, R. Kumar, S. Das, Process Intensification of Upgradation of Crude Oil and Vacuum Residue by Hydrodynamic Cavitation and Microwave Irradiation, Indian Chem. Eng. (2015). doi:10.1080/00194506.2015.1026949.

[6] C. Wan, R. Wang, W. Zhou, L. Li, Experimental study on viscosity reduction of heavy oil by hydrogen donors using a cavitating jet, RSC Adv. 9 (2019) 2509–2515. doi:10.1039/c8ra08087a.

[7] R. Terán Hilares, D.V. Kamoei, M.A. Ahmed, S.S. da Silva, J.I. Han, J.C. dos Santos, A new approach for bioethanol production from sugarcane bagasse using hydrodynamic cavitation assisted-pretreatment and column reactors, Ultrason. Sonochem. 43 (2018) 219–226. doi:10.1016/j.ultsonch.2018.01.016.

[8] L.F. Chuah, S. Yusup, A.R. Abd Aziz, A. Bokhari, J.J. Klemeš, M.Z. Abdullah, Intensification of biodiesel synthesis from waste cooking oil (Palm Olein) in a Hydrodynamic Cavitation Reactor: Effect of operating parameters on methyl ester conversion, Chem. Eng. Process. 95 (2015) 235–240. doi:10.1016/j.cep.2015.06.018.

[9] R. Terán Hilares, J.V. Ienny, P.F. Marcelino, M.A. Ahmed, F.A.F. Antunes, S.S. da Silva, J.C. dos Santos, Ethanol production in a simultaneous saccharification and fermentation process with interconnected reactors employing hydrodynamic cavitation-pretreated sugarcane bagasse as raw material, Bioresour. Technol. (2017). doi:10.1016/j.biortech.2017.06.159.

[10] A.K. Yadav, M.E. Khan, A. Pal, A.M. Dubey, Performance, emission and combustion characteristics of an Indica diesel engine operated with Yellow Oleander (Thevetia peruviana) oil biodiesel produced through hydrodynamic cavitation method, Int. J. Ambient Energy. 39 (2018) 365–371. doi:10.1080/01430750.2017.1303631.

[11] S. Bargole, S. George, V. Kumar Saharan, Improved rate of transesterification reaction in biodiesel synthesis using hydrodynamic cavitating devices of high throat perimeter to flow area ratios, Chem. Eng. Process. - Process Intensif. (2019). doi:10.1016/j.cep.2019.03.012.

[12] M.A. Kelkar, P.R. Gogate, A.B. Pandit, Intensification of esterification of acids for synthesis of biodiesel using acoustic and hydrodynamic cavitation, Ultrason. Sonochem. 15 (2008) 188–194. doi:10.1016/j.ultsonch.2007.04.003.

[13] A. V. Mohod, P.R. Gogate, G. Viel, P. Firmino, R. Giudici, Intensification of biodiesel production using hydrodynamic cavitation based on high speed homogenizer, Chem. Eng. J. (2017). doi:10.1016/j.cej.2017.02.011.

[14] G.L. Maddikeri, P.R. Gogate, A.B. Pandit, Intensified synthesis of biodiesel using

hydrodynamic cavitation reactors based on the interesterification of waste cooking oil, Fuel. 137 (2014) 285–292. doi:10.1016/j.fuel.2014.08.013.

[15] Y. Dai, Y. Qi, D. Zhao, H. Zhang, An oxidative desulfurization method using ultrasound/Fenton's reagent for obtaining low and/or ultra-low sulfur diesel fuel, Fuel Process. Technol. 89 (2008) 927–932. doi:10.1016/j.fuproc.2008.03.009.

[16] N.B. Suryawanshi, V.M. Bhandari, L.G. Sorokhaibam, V. V. Ranade, Investigating Adsorptive Deep Desulfurization of Fuels Using Metal-Modified Adsorbents and Process Intensification by Acoustic Cavitation, Ind. Eng. Chem. Res. 58 (2019) 7593–7606. doi:10.1021/acs.iecr.8b04043.

[17] N.S. More, P.R. Gogate, Intensified desulfurization of simulated crude diesel containing thiophene using ultrasound and ultraviolet irradiation, Ultrason. Sonochem. 58 (2019) 104612. doi:10.1016/j.ultsonch.2019.104612.

[18] J.M. Ramos, J.A. Wang, S.O. Flores, L.F. Chen, N. Nava, J. Navarrete, J.M. Domínguez, J.A. Szpunar, Ultrasound-assisted synthesis and catalytic activity of mesostructured FeOx/SBA-15 and FeOx/Zr-SBA-15 catalysts for the oxidative desulfurization of model diesel, Catal. Today. (2018). doi:10.1016/j.cattod.2018.04.059.

[19] Z. Shayegan, M. Razzaghi, A. Niaei, D. Salari, M.T.S. Tabar, A.N. Akbari, Sulfur removal of gas oil using ultrasound-assisted catalytic oxidative process and study of its optimum conditions, Korean J. Chem. Eng. 30 (2013) 1751–1759. doi:10.1007/s11814-013-0097-5.

[20] F.A. Duarte, P.D.A. Mello, C.A. Bizzi, M.A.G. Nunes, E.M. Moreira, M.S. Alencar, H.N. Motta, V.L. Dressler, É.M.M. Flores, Sulfur removal from hydrotreated petroleum fractions using ultrasound-assisted oxidative desulfurization process, Fuel. (2011).

doi:10.1016/j.fuel.2011.01.030.

[21] M. Zhao, P. Han, X. Lu, Ultrasound assisted photocatalytic oxidative desulfurization of model diesel fuel, Pet. Sci. Technol. 36 (2018) 29–33. doi:10.1080/10916466.2017.1403447.

[22] H. Mei, B.W. Mei, T.F. Yen, A new method for obtaining ultra-low sulfur diesel fuel via ultrasound assisted oxidative desulfurization, Fuel. 82 (2003) 405–414. doi:10.1016/S0016-2361(02)00318-6.

[23] K. Desai, S. Bhatt, S. Dharaskar, A. Unnarkat, S. Sasikumar Jampa, M. Khalid, Butyl triphenyl phosphonium bromide as an effective catalyst for ultrasound assisted oxidative desulfurization process, Mater. Today Proc. (2020). doi:10.1016/j.matpr.2019.12.308.

[24] Y. Lin, L. Feng, X. Li, Y. Chen, G. Yin, W. Zhou, Study on ultrasound-assisted oxidative desulfurization for crude oil, Ultrason. Sonochem. 63 (2020) 104946. doi:10.1016/j.ultsonch.2019.104946.

[25] M.A.G. Nunes, P.A. Mello, C.A. Bizzi, L.O. Diehl, E.M. Moreira, W.F. Souza, E.C. Gaudino, G. Cravotto, E.M.M. Flores, Evaluation of nitrogen effect on ultrasound-assisted oxidative desulfurization process, Fuel Process. Technol. (2014). doi:10.1016/j.fuproc.2014.05.031.

[26] A. Afzalinia, A. Mirzaie, A. Nikseresht, T. Musabeygi, Ultrasound-assisted oxidative desulfurization process of liquid fuel by phosphotungstic acid encapsulated in a interpenetrating amine-functionalized Zn(II)-based MOF as catalyst, Ultrason. Sonochem. 34 (2017) 713–720. doi:10.1016/j.ultsonch.2016.07.006.

[27] D. Carnaroglio, E.C. Gaudino, S. Mantegna, E.M. Moreira, A. Vicente De Castro, E.M.M. Flores, G. Cravotto, Ultrasound-assisted oxidative desulfurization/denitrification of liquid

fuels with solid oxidants, Energy and Fuels. 28 (2014) 1854–1859. doi:10.1021/ef402431e.

[28] L. Wang, Y. Chen, L. Du, S. Li, H. Cai, W. Liu, Nickel-heteropolyacids supported on silica gel for ultra-deep desulfurization assisted by Ultrasound and Ultraviolet, Fuel. (2013). doi:10.1016/j.fuel.2012.06.021.

[29] M.W. Wan, T.F. Yen, Portable continuous ultrasound-assisted oxidative desulfurization unit for marine gas oil, Energy and Fuels. 22 (2008) 1130–1135. doi:10.1021/ef7006358.

[30] A. Akbari, M. Omidkhah, J.T. Darian, Investigation of process variables and intensification effects of ultrasound applied in oxidative desulfurization of model diesel over MoO 3/Al2O3 catalyst, Ultrason. Sonochem. 21 (2014) 692–705. doi:10.1016/j.ultsonch.2013.10.004.

[31] D. Margeta, K. Sertić-Bionda, L. Foglar, Ultrasound assisted oxidative desulfurization of model diesel fuel, Appl. Acoust. (2016). doi:10.1016/j.apacoust.2015.07.004.

[32] J.B. Bhasarkar, S. Chakma, V.S. Moholkar, Investigations in physical mechanism of the oxidative desulfurization process assisted simultaneously by phase transfer agent and ultrasound, Ultrason. Sonochem. 24 (2015) 98–106. doi:10.1016/j.ultsonch.2014.11.008.

[33] L.A. Gonzalez, P. Kracke, W.H. Green, J.W. Tester, L.M. Shafer, M.T. Timko, Oxidative desulfurization of middle-distillate fuels using activated carbon and power ultrasound, Energy and Fuels. 26 (2012) 5164–5176. doi:10.1021/ef201289r.

[34] Q. Tang, S. Lin, Y. Cheng, S. Liu, J.R. Xiong, Ultrasound-assisted oxidative desulfurization of bunker-C oil using tert-butyl hydroperoxide, Ultrason. Sonochem. (2013). doi:10.1016/j.ultsonch.2013.02.002.

[35] A.E.S. Choi, S. Roces, N. Dugos, M.W. Wan, Oxidation by H2O2 of bezothiophene and dibenzothiophene over different polyoxometalate catalysts in the frame of ultrasound and mixing assisted oxidative desulfurization, Fuel. (2016). doi:10.1016/j.fuel.2016.04.014.

[36] J.B. Bhasarkar, S. Chakma, V.S. Moholkar, Mechanistic features of oxidative desulfurization using sono-fenton-peracetic acid (ultrasound/Fe2+-CH3COOH-H 2O2) system, Ind. Eng. Chem. Res. 52 (2013) 9038–9047. doi:10.1021/ie400879j.

[37] C. Zhou, Y. Wang, X. Huang, Y. Wu, J. Chen, Optimization of ultrasonic-assisted oxidative desulfurization of gasoline and crude oil, Chem. Eng. Process. - Process Intensif. 147 (2020) 107789. doi:10.1016/j.cep.2019.107789.

[38] M.K. Bolla, H.A. Choudhury, V.S. Moholkar, Mechanistic features of ultrasound-assisted oxidative desulfurization of liquid fuels, Ind. Eng. Chem. Res. (2012). doi:10.1021/ie300807a.

[39] G. Abdi, M. Ashokkumar, A. Alizadeh, Ultrasound-assisted oxidative-adsorptive desulfurization using highly acidic graphene oxide as a catalyst-adsorbent, Fuel. (2017). doi:10.1016/j.fuel.2017.09.024.

[40] S.H. Ammar, Y.S. Kareem, M.S. Mohammed, Catalytic-oxidative/adsorptive denitrogenation of model hydrocarbon fuels under ultrasonic field using magnetic reduced graphene oxide-based phosphomolybdic acid (PMo-Fe3O4/rGO), Ultrason. Sonochem. 64 (2020) 105050. doi:10.1016/j.ultsonch.2020.105050.

[41] G. Song, D.H. Wang, Z. Zhang, M. Liu, Q. Xu, D.Z. Zhao, A novel ultrasonic-assisted method for enhanced yield of light oil in the thermal cracking of residual oil, Ultrason. Sonochem. (2018). doi:10.1016/j.ultsonch.2018.05.029.

[42] D. Montes, E.A. Taborda, M. Minale, F.B. Cortés, C.A. Franco, Effect of the NiO/SiO2

Nanoparticles-Assisted Ultrasound Cavitation Process on the Rheological Properties of Heavy Crude Oil: Steady State Rheometry and Oscillatory Tests, Energy and Fuels. 33 (2019) 9671–9680. doi:10.1021/acs.energyfuels.9b02288.

[43] L.M. Mirzoeva, S.G. Yunusov, A.Z. Aliyeva, N.K. Andryushenko, Application of Ultrasonic Cavitation in the Etherification Reaction of a Coker Naphtha Narrow-Cut Fraction with Ethanol, Pet. Chem. 58 (2018) 671–675. doi:10.1134/S0965544118080169.

[44] Q. Fan, G. Bai, Q. Liu, Y. Sun, W. Yuan, S. Wu, X.M. Song, D.Z. Zhao, The ultrasound thermal cracking for the tar-sand bitumen, Ultrason. Sonochem. 50 (2019) 354–362. doi:10.1016/j.ultsonch.2018.09.035.

[45] R. Gopinath, A.K. Dalai, J. Adjaye, Effects of ultrasound treatment on the upgradation of heavy gas oil, Energy and Fuels. 20 (2006) 271–277. doi:10.1021/ef050231x.

[46] P. Kaushik, A. Kumar, T. Bhaskar, Y.K. Sharma, D. Tandon, H.B. Goyal, Ultrasound cavitation technique for up-gradation of vacuum residue, Fuel Process. Technol. (2012). doi:10.1016/j.fuproc.2011.09.005.

[47] B. Khodaei, M.A. Sobati, S. Shahhosseini, Optimization of ultrasound-assisted oxidative desulfurization of high sulfur kerosene using response surface methodology (RSM), Clean Technol. Environ. Policy. 18 (2016) 2677–2689. doi:10.1007/s10098-016-1186-z.

[48] J. Cui, Z. Zhang, X. Liu, L. Liu, J. Peng, Studies on viscosity reduction and structural change of crude oil treated with acoustic cavitation, Fuel. 263 (2020) 116638. doi:10.1016/j.fuel.2019.116638.

[49] C. Shi, W. Yang, J. Chen, X. Sun, W. Chen, H. An, Y. Duo, M. Pei, Application and mechanism of ultrasonic static mixer in heavy oil viscosity reduction, Ultrason. Sonochem. (2017). doi:10.1016/j.ultsonch.2017.02.027.

[50] F. Chemat, I. Grondin, P. Costes, L. Moutoussamy, A.S.C. Sing, J. Smadja, High power ultrasound effects on lipid oxidation of refined sunflower oil, Ultrason. Sonochem. 11 (2004) 281–285. doi:10.1016/j.ultsonch.2003.07.004.

[51] C. Stavarache, M. Vinatoru, R. Nishimura, Y. Maeda, Fatty acids methyl esters from vegetable oil by means of ultrasonic energy, Ultrason. Sonochem. 12 (2005) 367–372. doi:10.1016/j.ultsonch.2004.04.001.

[52] S.S. Kashyap, P.R. Gogate, S.M. Joshi, Ultrasound assisted synthesis of biodiesel from karanja oil by interesterification: Intensification studies and optimization using RSM, Ultrason. Sonochem. 50 (2019) 36–45. doi:10.1016/j.ultsonch.2018.08.019.

[53] A. Fallah Kelarijani, N. Gholipour Zanjani, A. Kamran Pirzaman, Ultrasonic Assisted Transesterification of Rapeseed Oil to Biodiesel Using Nano Magnetic Catalysts, Waste and Biomass Valorization. (2019). doi:10.1007/s12649-019-00593-1.

[54] I. Korkut, M. Bayramoglu, Selection of catalyst and reaction conditions for ultrasound assisted biodiesel production from canola oil, Renew. Energy. 116 (2018) 543–551. doi:10.1016/j.renene.2017.10.010.

[55] A. Nikseresht, A. Daniyali, M. Ali-Mohammadi, A. Afzalinia, A. Mirzaie, Ultrasound-assisted biodiesel production by a novel composite of Fe(III)-based MOF and phosphotangestic acid as efficient and reusable catalyst, Ultrason. Sonochem. 37 (2017) 203–207. doi:10.1016/j.ultsonch.2017.01.011.

[56] S.M. Hingu, P.R. Gogate, V.K. Rathod, Synthesis of biodiesel from waste cooking oil using sonochemical reactors, Ultrason. Sonochem. 17 (2010) 827–832. doi:10.1016/j.ultsonch.2010.02.010.

[57] A. Sarve, S.S. Sonawane, M.N. Varma, Ultrasound assisted biodiesel production from

sesame (Sesamum indicum L.) oil using barium hydroxide as a heterogeneous catalyst: Comparative assessment of prediction abilities between response surface methodology (RSM) and artificial neural network (ANN), Ultrason. Sonochem. 26 (2015) 218–228. doi:10.1016/j.ultsonch.2015.01.013.

[58] J. Prakash Maran, B. Priya, Comparison of response surface methodology and artificial neural network approach towards efficient ultrasound-assisted biodiesel production from muskmelon oil, Ultrason. Sonochem. 23 (2015) 192–200. doi:10.1016/j.ultsonch.2014.10.019.

[59] P.B. Subhedar, P.R. Gogate, Ultrasound assisted intensification of biodiesel production using enzymatic interesterification, Ultrason. Sonochem. 29 (2016) 67–75. doi:10.1016/j.ultsonch.2015.09.006.

[60] S. Michelin, F.M. Penha, M.M. Sychoski, R.P. Scherer, H. Treichel, A. Valério, M. Di Luccio, D. de Oliveira, J.V. Oliveira, Kinetics of ultrasound-assisted enzymatic biodiesel production from Macauba coconut oil, Renew. Energy. 76 (2015) 388–393. doi:10.1016/j.renene.2014.11.067.

[61] M.V.P. Rocha, L.J.B.L. de Matos, L.P. de Lima, P.M. da S. Figueiredo, I.L. Lucena, F.A.N. Fernandes, L.R.B. Gonçalves, Ultrasound-assisted production of biodiesel and ethanol from spent coffee grounds, Bioresour. Technol. 167 (2014) 343–348. doi:10.1016/j.biortech.2014.06.032.

[62] W.W.S. Ho, H.K. Ng, S. Gan, Advances in ultrasound-assisted transesterification for biodiesel production, Appl. Therm. Eng. 100 (2016) 553–563. doi:10.1016/j.applthermaleng.2016.02.058.

[63] S. Manickam, V.N.D. Arigela, P.R. Gogate, Intensification of synthesis of biodiesel from

palm oil using multiple frequency ultrasonic flow cell, Fuel Process. Technol. 128 (2014) 388–393. doi:10.1016/j.fuproc.2014.08.002.

[64] U. Rashid, H.A. Rehman, I. Hussain, M. Ibrahim, M.S. Haider, Muskmelon (Cucumis melo) seed oil: A potential non-food oil source for biodiesel production, Energy. 36 (2011) 5632–5639. doi:10.1016/j.energy.2011.07.004.

[65] N.S. More, P.R. Gogate, Intensified approach for desulfurization of simulated fuel containing thiophene based on ultrasonic flow cell and oxidizing agents, Ultrason. Sonochem. 51 (2019) 58–68. doi:10.1016/j.ultsonch.2018.10.019.

[66] J. Bhasarkar, A.J. Borah, P. Goswami, V.S. Moholkar, Mechanistic analysis of ultrasound assisted enzymatic desulfurization of liquid fuels using horseradish peroxidase, Bioresour. Technol. (2015). doi:10.1016/j.biortech.2015.07.063.

[67] N.S. More, P.R. Gogate, Intensified desulfurization of simulated crude diesel containing thiophene using ultrasound and ultraviolet irradiation, Ultrason. Sonochem. (2019). doi:10.1016/j.ultsonch.2019.104612.

[68] G.L. Maddikeri, A.B. Pandit, P.R. Gogate, Ultrasound assisted interesterification of waste cooking oil and methyl acetate for biodiesel and triacetin production, Fuel Process. Technol. (2013). doi:10.1016/j.fuproc.2013.07.004.

[69] M.A. Kelkar, P.R. Gogate, A.B. Pandit, Intensification of esterification of acids for synthesis of biodiesel using acoustic and hydrodynamic cavitation, Ultrason. Sonochem. (2008). doi:10.1016/j.ultsonch.2007.04.003.

[70] N.B. Suryawanshi, V.M. Bhandari, L.G. Sorokhaibam, V. V. Ranade, A Non-catalytic Deep Desulphurization Process using Hydrodynamic Cavitation, Sci. Rep. (2016). doi:10.1038/srep33021.

[71] S. Baradaran, M.T. Sadeghi, Intensification of diesel oxidative desulfurization via hydrodynamic cavitation, Ultrason. Sonochem. (2019). doi:10.1016/j.ultsonch.2019.104698.

[72] K.B. Ansari, N.H. Loke, A.B. Pandit, V.G. Gaikar, R. Sivakumar, R. Kumar, S. Das, Process Intensification of Upgradation of Crude Oil and Vacuum Residue by Hydrodynamic Cavitation and Microwave Irradiation, Indian Chem. Eng. (2015). doi:10.1080/00194506.2015.1026949.

[73] R. Terán Hilares, D.V. Kamoei, M.A. Ahmed, S.S. da Silva, J.I. Han, J.C. dos Santos, A new approach for bioethanol production from sugarcane bagasse using hydrodynamic cavitation assisted-pretreatment and column reactors, Ultrason. Sonochem. 43 (2018) 219–226. doi:10.1016/j.ultsonch.2018.01.016.

[74] L.F. Chuah, S. Yusup, A.R. Abd Aziz, A. Bokhari, J.J. Klemeš, M.Z. Abdullah, Intensification of biodiesel synthesis from waste cooking oil (Palm Olein) in a Hydrodynamic Cavitation Reactor: Effect of operating parameters on methyl ester conversion, Chem. Eng. Process. Process Intensif. 95 (2015) 235–240. doi:10.1016/j.cep.2015.06.018.

[75] S. Bargole, S. George, V. Kumar Saharan, Improved rate of transesterification reaction in biodiesel synthesis using hydrodynamic cavitating devices of high throat perimeter to flow area ratios, Chem. Eng. Process. - Process Intensif. (2019). doi:10.1016/j.cep.2019.03.012.
